# Immunoglobulin E-virus phenotypes of infant bronchiolitis and risk of childhood asthma

**DOI:** 10.3389/fimmu.2023.1187065

**Published:** 2023-05-10

**Authors:** Ryohei Shibata, Zhaozhong Zhu, Tadao Ooka, Robert J. Freishtat, Jonathan M. Mansbach, Marcos Pérez-Losada, Ignacio Ramos-Tapia, Stephen Teach, Carlos A. Camargo, Kohei Hasegawa

**Affiliations:** ^1^ Department of Emergency Medicine, Massachusetts General Hospital, Harvard Medical School, Boston, MA, United States; ^2^ Department of Health Science, University of Yamanashi, Yamanashi, Japan; ^3^ Center for Genetic Medicine Research, Children’s National Research Institute, Washington, DC, United States; ^4^ Division of Emergency Medicine, Children’s National Hospital, Washington, DC, United States; ^5^ Department of Pediatrics, The George Washington University School of Medicine and Health Sciences, Washington, DC, United States; ^6^ Department of Pediatrics, Boston Children’s Hospital, Harvard Medical School, Boston, MA, United States; ^7^ Department of Biostatistics and Bioinformatics, Computational Biology Institute, The George Washington University, Washington, DC, United States; ^8^ Microbial Data Science Laboratory, Center for Bioinformatics and Integrative Biology, Universidad Andres Bello, Santiago, Chile; ^9^ Center for Translational Research, Children’s National Research Institute, Washington, DC, United States

**Keywords:** asthma, bronchiolitis, immunoglobulin E, microRNA, mRNA, phenotyping, RSV (respiratory syncytial virus), rhinovirus (RV)

## Abstract

**Background:**

Bronchiolitis is the leading cause of infant hospitalization in U.S. and is associated with increased risk for childhood asthma. Immunoglobulin E (IgE) not only plays major roles in antiviral immune responses and atopic predisposition, but also offers a potential therapeutic target.

**Objective:**

We aimed to identify phenotypes of infant bronchiolitis by using total IgE (tIgE) and virus data, to determine their association with asthma development, and examine their biological characteristics.

**Methods:**

In a multicenter prospective cohort study of 1,016 infants (age <1 year) hospitalized for bronchiolitis, we applied clustering approaches to identify phenotypes by integrating tIgE and virus (respiratory syncytial virus [RSV], rhinovirus [RV]) data at hospitalization. We examined their longitudinal association with the risk of developing asthma by age 6 years and investigated their biological characteristics by integrating the upper airway mRNA and microRNA data in a subset (n=182).

**Results:**

In infants hospitalized for bronchiolitis, we identified 4 phenotypes: 1) tIgE^low^virus^RSV-high^, 2) tIgE^low^virus^RSV-low/RV^, 3) tIgE^high^virus^RSV-high^, and 4) tIgE^high^virus^RSV-low/RV^ phenotypes. Compared to phenotype 1 infants (resembling “classic” bronchiolitis), phenotype 4 infants (tIgE^high^virus^RSV-low/RV^) had a significantly higher risk for developing asthma (19% vs. 43%; adjOR, 2.93; 95% CI, 1.02–8.43; *P*=.046). Phenotypes 3 and 4 (tIgE^high^) had depleted type I interferon and enriched antigen presentation pathways; phenotype 4 also had depleted airway epithelium structure pathways.

**Conclusions:**

In this multicenter cohort, tIgE-virus clustering identified distinct phenotypes of infant bronchiolitis with differential risks of asthma development and unique biological characteristics.

## Introduction

Bronchiolitis is the leading cause of infant hospitalization in the U.S., accounting for 110,000 hospitalizations annually (1). Bronchiolitis also puts infants at increased risk for subsequent morbidity—~30% of infants hospitalized for bronchiolitis develop asthma (2). While bronchiolitis has traditionally been considered a single disease with a similar mechanism (3), growing evidence supports disease heterogeneity (4–6). Indeed, recent studies have reported subgroups (phenotypes and endotypes) of infant bronchiolitis with different risks of asthma by using clinical (7, 8) and molecular (9–11) data. Yet, no subgroup-specific approach for asthma prevention has been developed in this high-risk population.

In childhood asthma, immunoglobulin E (IgE) plays an important role (12) and is a major therapeutic target (13, 14). Research has also shown that, among infants (age <12 months) with parental atopy (15) and hospitalized for bronchiolitis (16), IgE-mediated sensitization is associated with a higher risk of developing asthma. Additionally, considering that IgE-mediated sensitization is uncommon in early infancy (16), a recent study of infant bronchiolitis has demonstrated the relationship of a higher total IgE (tIgE) level with an increased risk of developing asthma (17). Furthermore, research has found that IgE impairs innate antiviral immune responses (e.g., type I interferon) through cross-linking of high-affinity IgE receptor (FcϵRI) (18–20), thereby potentially leading to severe viral infection and subsequent asthma development. Despite the public health and research significance, little is known about the relationship among IgE, viral infection, immune responses in infants with bronchiolitis, and their integrated contributions to the subsequent development of asthma.

To address this knowledge gap, we analyzed data from a multicenter cohort study of infants hospitalized for bronchiolitis to 1) identify tIgE-virus phenotypes, 2) examine their longitudinal associations with asthma development, and 3) determine their biological characteristics by integrating upper airway mRNA and microRNA (miRNA) data. A better understanding of tIgE-driven phenotypes may inform early-life prevention strategies (e.g., anti-IgE antibody) against the development of asthma.

## Methods

### Study design, setting, and participants

We analyzed data from the 35th Multicenter Airway Research Collaboration (MARC-35) study—a multicenter prospective cohort study. Details of the study design, setting, participants, data collection, testing, and statistical analysis may be found in the **Supplementary Methods**. Briefly, investigators enrolled infants (<12 months) hospitalized with attending physician-diagnosis of bronchiolitis at 17 sites across 14 U.S. states (**Table E1**) in 2011–2014. Bronchiolitis was defined by the American Academy of Pediatrics (AAP) guidelines—acute respiratory illness with some combination of rhinitis, cough, tachypnea, wheezing, crackles, and retractions, regardless of previous breathing problem episodes (3). We excluded infants with a known heart-lung disease, immunodeficiency, immunosuppression, or gestational age < 32 weeks. All patients were treated at the discretion of the treating physician. The institutional review board at each participating hospital approved the study with written informed consent obtained from the parent or guardian.

### Data collection

Clinical data (demographic characteristics; medical, environmental, and family history; and details of the bronchiolitis course) were collected *via* structured interview and chart review using a standardized protocol (21). All data were reviewed at the EMNet Coordinating Center (Boston, MA, U.S.), and site investigators were queried about missing data and discrepancies identified by manual data checks. Serum, nasopharyngeal, and nasal specimens were collected using standard protocols (21) within 24 hours of hospitalization. Serum tIgE was measured by using ImmunoCAP Total IgE (Thermo Fisher Scientific, Waltham, MA, USA); the range of this assay is 2 to 5,000 kU/L. Upper airway (nasopharyngeal and nasal) specimens were tested for respiratory viruses (e.g., respiratory syncytial virus [RSV] and rhinovirus [RV]) by real-time polymerase chain reaction assays (16), mRNA profiling by RNA sequencing (RNA-seq), and miRNA profiling by small RNA-seq.

### Nasopharyngeal mRNA profiling

The details of RNA extraction, RNA-seq, and quality control are described in **Supplementary Methods** and previous studies (9). Briefly, after applying total RNA extraction, DNase treatment, and rRNA to randomly-selected 244 specimens, we performed RNA-seq with a NovaSeq6000 (Illumina, San Diego, CA, U.S.) using an S4 100bp PE Flowcell (Illumina). After quality control, all RNA-seq samples had high sequence coverage (mean of 8,067,019 pair-end reads/sample). We estimated transcript abundances in Salmon (22) using the human genome (hg38) and the mapping-based mode.

### Nasal miRNA profiling

The details of RNA extraction, small RNA-seq, and quality control are described in **Supplementary Methods**. Briefly, after total RNA extraction, DNase treatment, and rRNA reduction, we used 624 specimens with sufficient RNA quantity and quality to perform small RNA-seq with a NovaSeq6000 using an S2 50bp PE Flowcell (Illumina). All small RNA-seq samples had sufficient sequence depth (mean of 26,736,490 pair-end reads/sample) to obtain a high sequence coverage. From clean small RNA-seq reads, we estimated miRNA detection and abundance by sMETASeq (23). We mapped trimmed reads against human miRNA sequences from miRBase V22 (24).

### Outcome measure

The outcome of interest was the development of asthma by age 6 years. Asthma was defined using a commonly used epidemiologic definition (25): physician diagnosis of asthma, with either asthma medication use (e.g., inhaled bronchodilators and inhaled corticosteroids) or asthma-related symptoms (e.g., wheezing and nocturnal cough) during the year before the evaluation at age 6 years.

### Statistical analysis

**Figure 1:**
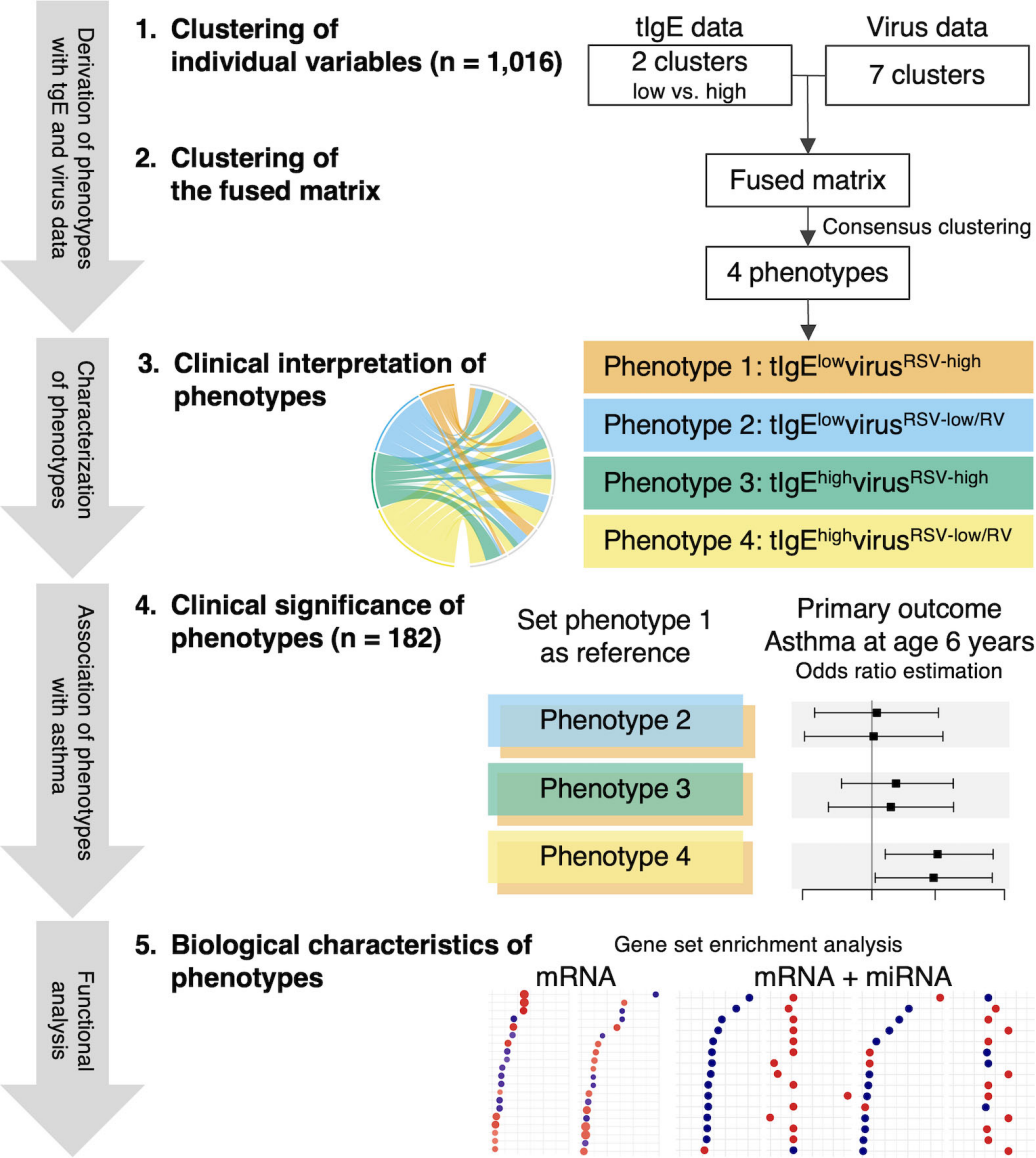
Analytic workflow of phenotyping with total immunoglobulin E and virus data. 1. Clustering of individual variables. By using the multicenter prospective cohort (MARC-35) data of 1,016 infants (age <1 year) hospitalized for bronchiolitis, we first identified mutually exclusive clusters for each of the total immunoglobulin E (tIgE) and virus datasets. We generated the low and high tIgE clusters by the median value and identified the 7 virus clusters by computing a Gower distance, followed by applying consensus clustering algorithms to the distance matrices. 2. Clustering of the fused matrix. By integrating these derived clusters from the tIgE and virus datasets, we generated a fused matrix and computed a Gower distance. We identified 4 mutually exclusive tIgE-virus phenotypes by applying a consensus clustering algorithm to the distance matrix. To select an optimal number of profiles, we used a combination of consensus matrix and consensus cumulative distribution function, in addition to phenotype size and clinical plausibility. 3. Clinical interpretation of phenotypes. To interpret the clinical characteristics of the 4 phenotypes, we developed chord diagrams on major clinical and virus factors. 4. Clinical significance of phenotypes. Of MARC-35, 182 infants with nasopharyngeal mRNA and nasal microRNA (miRNA) data at enrollment with the asthma outcome data were selected as the analytic cohort. To examine the clinical significance of the 4 phenotypes, we determined the longitudinal relationship of the phenotypes with the risk of developing asthma by constructing logistic regression models. 5. Biological characteristics of phenotypes. To examine the difference in biological characteristics between the phenotypes, we first conducted a functional pathway analysis by using the nasopharyngeal mRNA data. Next, we conducted a functional pathway analysis by integrating the mRNA data with the nasal miRNA data. RSV, respiratory syncytial virus; RV, rhinovirus.

The analytic workflow is summarized in **Figure 1**. The details of the statistical analysis can be found in the **Supplementary Methods**. Briefly, we first identified mutually exclusive clusters for each of the tIgE and virus datasets collected at the index hospitalization. For the tIgE data, we generated the low and high tIgE clusters by using the median value. In contrast to tIgE clustering with the use of median cut-off value, for the virus data (including the genomic load [i.e., cycle threshold value] of RSV and RV), we computed a Gower distance and derived the virus clusters by using a consensus clustering algorithm with partitioning around medoids (PAM) method. To choose an optimal number of the virus clusters, we used a combination of separations of the consensus matrix and relative change of the area under the cumulative distribution function (CDF) curve, in addition to the cluster size and clinical plausibility (16, 26). Second, we combined the tIgE and virus clusters to derive a fused matrix, computed a Gower distance, and identified 4 mutually exclusive phenotypes by using a consensus clustering algorithm with PAM method. Third, to interpret the clinical characteristics of the phenotypes, we developed chord diagrams. Fourth, we determined the longitudinal association of the phenotypes with the asthma risk. Of the 1,016 MARC-35 infants, we used 182 infants with mRNA, miRNA, and asthma outcome data as the analytic cohort. Then, we constructed unadjusted logistic regression and multivariable mixed-effects logistic regression models. In the multivariable mixed-effects model, we adjusted for site effects and patient-level potential confounders (i.e., age, sex, parental history of asthma, prematurity [<37 weeks], pre-hospitalization use of inhaled and/or systemic corticosteroids) based on clinical plausibility and *a priori* knowledge (27, 28). Fifth, we examined the difference in biological characteristics between the derived phenotypes by using the nasopharyngeal mRNA data. We conducted differential expression gene analysis between the phenotypes and Gene Set Enrichment Analysis (GSEA) (29) based on Biological Processes in Gene Ontology (false discovery rate [FDR] <0.10). Sixth, we also examined the difference in biological characteristics by integrating the mRNA and miRNA data. As with the mRNA data, we conducted differential expression miRNA analysis and miRNA set enrichment analysis based on Biological Processes in Gene Ontology derived from the miRNA-target databases, miRTarBase 8.0 (30). We identified pathways that are enriched in the mRNA data and depleted in the miRNA data or that are depleted in the mRNA data and enriched in the miRNA data (FDR <0.10).

In the sensitivity analysis, we first computed E-values to determine the robustness of causal inference to potential unmeasured confounding. We next examined the phenotype-outcome associations after excluding infants with a previous history of breathing problems. We also examined the robustness of the phenotype-outcome associations under different definitions of exposure (31) by repeating the analysis using a different number of phenotypes. We conducted statistical analyses using R version 4.1.2 (R Foundation, Vienna, Austria). All P-values were two-tailed, with *p* < 0.05 considered statistically significant. We corrected for multiple hypothesis testing using the Benjamini-Hochberg FDR with FDR <0.10 considered statistically significant.

## Results

Among 1,016 infants hospitalized for bronchiolitis, the median age was 3 (interquartile range 2–6) months, 60% were male, and 42% were non-Hispanic white. Overall, 81% had RSV, 21% had RV, and 16% underwent intensive treatment use (the use of positive pressure ventilation and/or admission to intensive care unit) during the index hospitalization (**Table E2**).

### Integrated clustering of the tIgE and virus data identified distinct phenotypes among infants with bronchiolitis

First, the low and high tIgE clusters by the median value were generated. Second, by applying clustering approaches to the virus data, a 7-class model provided an optimal fit (**Figure E1**). Next, by integrating these cluster data using the consensus clustering approach, the combination of the consensus matrix and relative change of the area under the CDF curve in addition to phenotype size (n = 225–283) and clinical plausibility found that a 4-class model provided an optimal fit (**Figure E2**) with the 4 tIgE-virus phenotypes called 1, 2, 3, and 4. The 4 distinct phenotypes were 1) tIgE^low^virus^RSV-high^ (23%), 2) tIgE^low^virus^RSV-low/RV^ (27%), 3) tIgE^high^virus^RSV-high^ (22%); and 4) tIgE^high^virus^RSV-low/RV^ (28%; **Table E2**; **Figure 1**).

**Figure 2:**
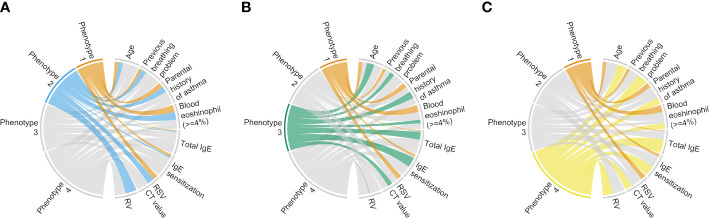
Clinical and virus characteristics of infants hospitalized for bronchiolitis in MARC-35, according to phenotypes. To interpret clinical and virus characteristics of the 4 phenotypes, we constructed chord diagrams that represent the comparison between phenotypes **(A)** 1 and 2, **(B)** 1 and 3, and **(C)** 1 and 4. Ribbons connect each of the phenotypes (phenotypes 1–4) with major clinical and virus characteristics. The width of the ribbon represents the proportion of infants or the mean value within the phenotypes who have the corresponding clinical or virus characteristic, which was scaled to a total of 100% or 1. Of the clinical and virus characteristics, we selected 8 characteristics that may be related to “classic” bronchiolitis and coordinated the presence or increase of those in the same direction. Hence, the phenotype with the narrower bundle of ribbons resembles “classic” bronchiolitis (phenotype 1). CT, cycle threshold; IgE, immunoglobulin E; RSV, respiratory syncytial virus; RV, rhinovirus.

Descriptively, infants with a phenotype 1 or 2 were similarly characterized by young age at the index hospitalization with a low proportion of previous breathing problems, tIgE level, and proportion of IgE sensitization (**Table E2**; **Figure 2A**). Regarding the virus data, infants with a phenotype 1 were characterized by RSV infection (with a high genomic load), while those with a phenotype 2 were characterized by RSV (with a low genomic load) and RV infection. As the phenotype 1 clinically resembled “classic” bronchiolitis (3), this group served as the reference group for the following analyses. In contrast, infants with a phenotype 3 or 4 were similarly characterized by older age at the index hospitalization with a high tIgE level and proportion of IgE sensitization (**Table E2**; **Figures 2B, C**). Infants with a phenotype 3 were characterized by a low proportion of previous breathing problems and RSV infection (with a high genomic load), while those with a phenotype 4 were characterized by a high proportion of previous breathing problems and RSV (with a low genomic load) and RV infection.

### tIgE-virus phenotypes had differential risks for developing asthma

**Figure 3:**
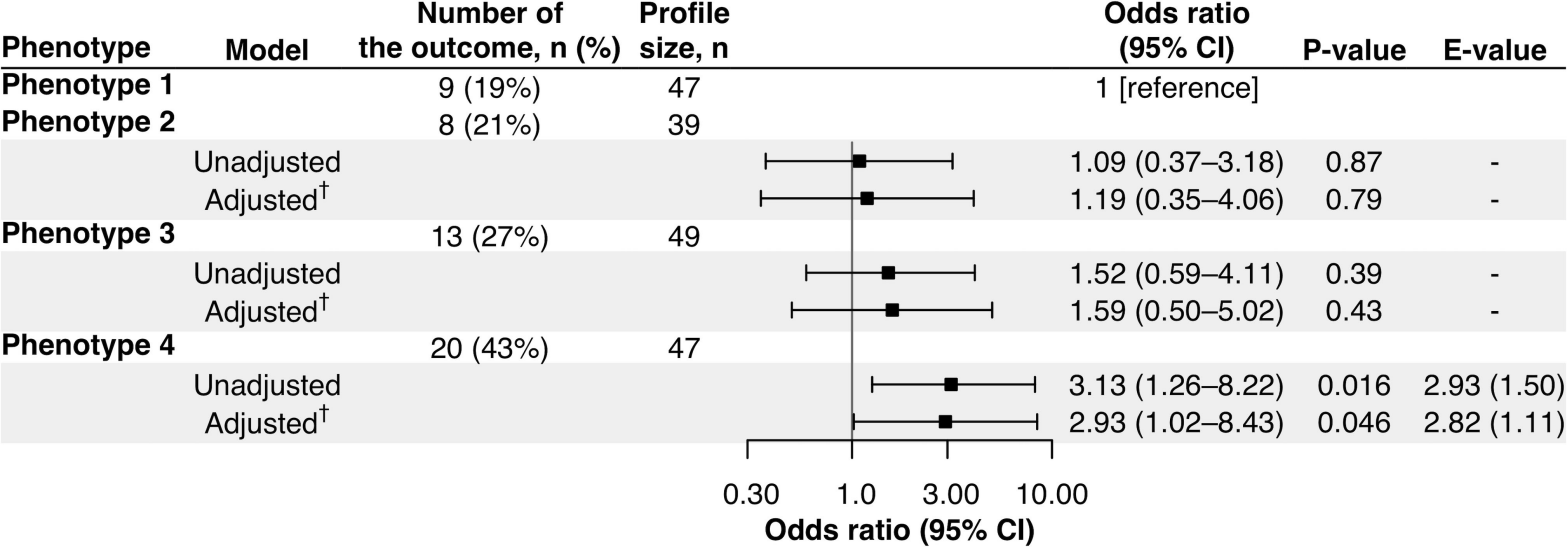
Association of phenotypes of infant bronchiolitis with risk of developing childhood asthma. To examine the association of bronchiolitis phenotypes (phenotype 1 as the reference) with the risk of developing asthma, logistic regression models were constructed. ^†^The E-value (with its lower 95% confidence interval [CI] bound) represents how strongly unmeasured confounder(s) represents how strongly a set of unmeasured confounders would be associated with the exposure and outcome to fully eliminate the observed association. ^‡^Multivariable mixed-effects logistic regression model accounting for patient clustering by site and adjusted for potential confounders (i.e., age, sex, parental history of asthma, prematurity [<37 weeks], previous history of breathing problems, and pre-hospitalization use of inhaled and/or systemic corticosteroids).

Of the 1,016 infants, the following analyses focused on 182 infants with the nasopharyngeal mRNA and nasal miRNA data at enrollment with the asthma outcome data at age 6 years. The analytic and non-analytic cohorts did not differ in the clinical characteristics (*p* ≥ 0.05; **Table E3**), except for the proportion of daycare use, oxygen saturation at the presentation, and proportion of RSV infection. Furthermore, the significantly different characteristics among the 4 phenotypes were almost consistent between MARC-35 and the analytic cohort (**Table 1**). The identified phenotypes had differential risks for developing asthma. Compared with phenotype 1, phenotype 4 (tIgE^high^virus^RSV-low/RV^) had a significantly higher risk of developing asthma (19% vs. 43%; adjusted odds ratio [adjOR] 2.93; 95% confidence interval [CI], 1.02–8.43; *p* = 0.046; E-value = 2.82; **Figure 3**). In contrast, the risk of asthma was not significantly different in phenotype 2 or 3 (**Figure 3**).

**Table 1:** Baseline patient characteristics and clinical course of infants hospitalized for bronchiolitis in the analytic cohort, according to 4 phenotypes.

Variables	Overall (n = 182; 100%)	Phenotype 1 (tIgE^low^ virus^RSV-high^) (n = 47; 26%)	Phenotype 2 (tIgE^low^ Virus^RSV-low/RV^) (n = 39; 21%)	Phenotype 3 (tIgE^high^ virus^RSV-high^) (n = 49; 27%)	Phenotype 4 (tIgE^high^ Virus^RSV-low/RV^) (n = 47; 26%)	P-value^†^
Demographics						
Age (month), median (IQR)	3 (2–6)	2 (1–4)	2 (1–4)	5 (2–7)	5 (2–8)	<.001^‡^
Male sex	104 (57)	27 (57)	23 (59)	24 (49)	30 (64)	.52
Race/ethnicity						<.001
Non-Hispanic white	75 (41)	23 (49)	23 (59)	21 (43)	8 (17)	
Non-Hispanic black	45 (25)	8 (17)	4 (10)	14 (29)	19 (40)	
Hispanic	56 (31)	16 (34)	9 (23)	11 (22)	20 (43)	
Other	6 (3)	0 (0)	3 (8)	3 (6)	0 (0)	
C-section delivery	62 (34)	18 (38)	9 (23)	20 (41)	15 (32)	.37
Prematurity (32–36.9 weeks)	31 (17)	9 (19)	4 (10)	11 (22)	7 (15)	.46
History of eczema	24 (13)	4 (9)	2 (5)	8 (16)	10 (21)	.10
Previous breathing problems (count)						.046
0	155 (85)	41 (87)	37 (95)	44 (90)	33 (70)	
1	20 (11)	5 (11)	1 (3)	4 (8)	10 (21)	
≥2	7 (4)	1 (2)	1 (3)	1 (2)	4 (9)	
Lifetime corticosteroid use^§^	31 (17)	7 (15)	7 (18)	6 (12)	11 (23)	.51
Pre-hospitalization corticosteroid use^¶^	19 (10)	3 (6)	6 (15)	5 (10)	5 (11)	.60
Ever attended daycare	54 (30)	11 (23)	12 (31)	17 (35)	14 (30)	.68
Cigarette smoke exposure at home	27 (15)	8 (17)	3 (8)	7 (14)	9 (19)	.48
Parental history of eczema	36 (20)	7 (15)	7 (18)	12 (24)	10 (21)	.67
Parental history of asthma	64 (35)	16 (34)	14 (36)	17 (35)	17 (36)	.99
Clinical presentation at index hospitalization						
Weight (kg), median (IQR)	6.2 (4.6–8.0)	5.2 (4.3–6.2)	5.5 (4.4–7.0)	7.2 (5.3–8.5)	7.1 (5.0–8.6)	<.001^‡^
Respiratory rate (per minute), median (IQR)	48 (40–60)	48 (40–56)	48 (40–60)	46 (38–60)	48 (41–60)	.74^‡^
Oxygen saturation						.41
<90%	11 (6)	5 (11)	2 (5)	2 (4)	2 (4)	
90–93.9%	20 (11)	6 (13)	5 (13)	8 (16)	1 (2)	
≥94%	148 (81)	36 (77)	31 (79)	38 (78)	43 (91)	
Blood testing						
Blood eosinophilia (≥4%)	15 (8)	5 (11)	3 (8)	3 (6)	4 (9)	.91
tIgE (kU/L), median (IQR)	4.8 (1.9–15.5)	1.9 (1.9–2.7)	1.9 (1.9–2.4)	10.9 (6.6–22.4)	19.9 (8.1–40.9)	<.001^‡^
Allergic (specific IgE) sensitization	45 (25)	4 (9)	2 (5)	17 (35)	22 (47)	<.001
Aeroallergen sensitization	1 (1)	0 (0)	0 (0)	0 (0)	1 (2)	.41
Food sensitization	44 (24)	4 (9)	2 (5)	17 (35)	21 (45)	<.001
Viral testing						
RSV	167 (92)	47 (100)	35 (90)	49 (100)	36 (77)	<.001
RSV cycle threshold value	22 (20–25)	21 (19–22)	26 (23–27)	21 (19–22)	26 (25–30)	<.001^‡^
RV	37 (20)	0 (0)	17 (44)	0 (0)	20 (43)	<.001
Other pathogens**^††^**	38 (21)	6 (13)	7 (18)	10 (20)	15 (32)	.14
Clinical course						
Positive pressure ventilation use^‡‡^	10 (5)	3 (6)	4 (10)	0 (0)	3 (6)	.20
Intensive treatment use^§§^	26 (14)	5 (11)	6 (15)	6 (12)	9 (19)	.65

Data are the number (percentage) of children unless otherwise indicated. Percentages may not equal 100 because of rounding and missingness. All data are collected, unless otherwise indicated.

IgE, immunoglobulin E; IQR, interquartile range; RSV, respiratory syncytial virus; RV, rhinovirus; tIgE, total immunoglobulin E.

**^†^** Tested by the chi-square test, unless otherwise indicated.

^‡^Tested by the Kruskal-Wallis test.

^§^Defined as the use of inhaled and/or systemic corticosteroids before the index hospitalization.

^¶^Defined as the use of inhaled and/or systemic corticosteroids for breathing problems that caused the index hospitalization.

**^††^** Adenovirus, bocavirus, Bordetella pertussis, enterovirus, human coronavirus NL63, OC43, 229E, or HKU1, human metapneumovirus, influenza A or B virus, Mycoplasma pneumoniae, and parainfluenza virus 1–3.

^‡‡^Defined as the use of invasive and/or non-invasive mechanical ventilation (e.g., continuous positive airway pressure ventilation) during the index hospitalization.

^§§^Defined as the use of positive pressure ventilation and/or admission to intensive care unit

### tIgE-virus phenotypes had distinct biological characteristics

**Figure 4:**
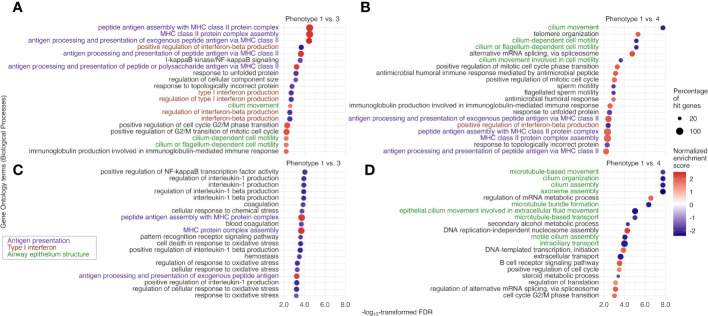
Between-phenotype differences (1 vs. 3 and 4) in nasopharyngeal mRNA pathways among infants hospitalized for bronchiolitis. To examine the difference in the biological characteristics between phenotypes (1 [the reference] vs. 3 and 4 [i.e., tIgE^high^ phenotypes]), we applied the gene set enrichment analysis based on Biological Processes in Gene Ontology to the nasopharyngeal mRNA data. We identified enriched (normalized enrichment score [NES] ≥ 0 and false discovery rate [FDR] <.10) or depleted (NES <0 and FDR <.10) pathways. Of the identified pathways shared between the phenotype 1 vs. 3 and 1 vs. 4 comparisons, we selected the 20 pathways with the lowest FDR for **(A)** phenotype 1 vs. 3 and **(B)** phenotype 1 vs. 4. Likewise, of the identified pathways unique to the phenotype **(C)** 1 vs. 3 and **(D)** 1 vs. 4 comparisons, we selected the 20 pathways with the lowest FDR. MHC, major histocompatibility complex; NF-kappaB, nuclear factor κ-light-chain-enhancer of activated B cells.

**Figure 5:**
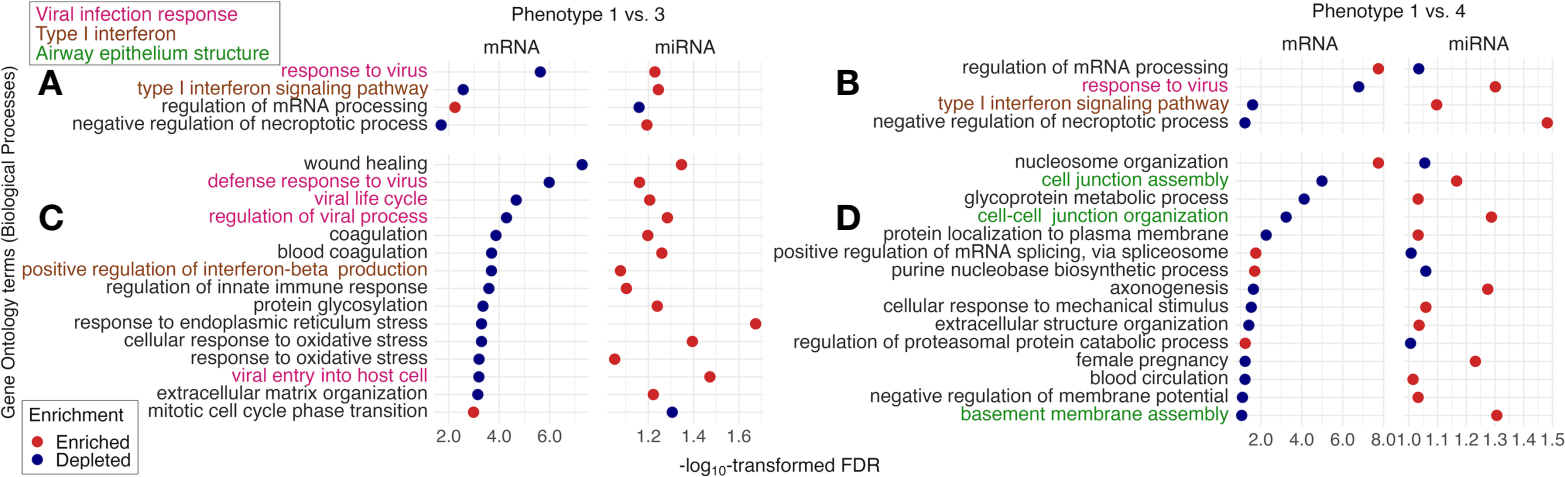
Between-phenotype differences (1 vs. 3 and 4) in nasopharyngeal mRNA pathways and nasal microRNA pathways among infants hospitalized for bronchiolitis. To examine the difference in the biological characteristics between phenotypes (1 [the reference] vs. 3 and 4 [i.e., tIgE^high^ phenotypes]), we applied the gene set enrichment analysis based on Biological Processes in Gene Ontology to the nasopharyngeal mRNA data and the nasal microRNA (miRNA) data. We identified the pathways “enriched in the mRNA data and depleted in the miRNA data” or “depleted in the mRNA data and enriched in the miRNA data” (false discovery rate [FDR] <.10), accounting for suppressive nature of miRNA to mRNA. The identified pathways shared between phenotype 1 vs. 3 and 1 vs. 4 comparisons are shown in **(A, B)**. Of the identified pathways unique to **(C)** phenotype 1 vs. 3 and **(D)** phenotype 1 vs. 4, we selected the 15 pathways with the lowest FDR in the mRNA data.

The tIgE-virus phenotypes of infant bronchiolitis had distinct biological characteristics. With the mRNA data, compared with phenotype 1, phenotype 2, 3, and 4 had different gene expression signatures (**Figure E3**) and significantly enriched or depleted pathways (FDR < 0.10; **Figures 4**, **E4**). Of the identified pathways, phenotypes 3 and 4 (i.e., tIgE^high^ phenotypes) shared the antigen presentation, type I interferon, and airway epithelium structure pathways (**Figures 4A, B**). Phenotype 3 (tIgE^high^virus^RSV-high^) had enriched antigen presentation pathways (e.g., peptide antigen assembly with major histocompatibility complex [MHC] class II protein complex) and depleted type I interferon pathways (e.g., positive regulation of interferon-beta production; **Figures 4A, C**). In contrast, phenotype 4 (tIgE^high^virus^RSV-low/RV^) had depleted airway epithelium structure pathways (e.g., cilium movement; **Figures 4B, D**). Likewise, with the miRNA data, phenotypes 2, 3, and 4 had different miRNA expression signatures (**Figure E5**). By integrating the significantly enriched or depleted miRNA pathways (FDR < 0.10) with the identified mRNA pathways, we determined the enriched or depleted pathways in both data (**Figures 5**, **E6**). Similar to the results of mRNA data, phenotypes 3 and 4 shared 4 common pathways (e.g., response to virus and type I interferon signaling pathway; **Figures 5A, B**). Likewise, phenotype 3 had depleted virus infection response (e.g., defense response to virus; **Figure 5C**) and type I interferon pathways (e.g., positive regulation of interferon-beta production), while phenotype 4 had depleted airway epithelium structure pathways (e.g., cell junction assembly; **Figure 5D**).

### Sensitivity analysis

In the analysis excluding infants with a previous history of breathing problems with limited statistical power (n=155), the phenotype-asthma associations did not show statistical significance in the unadjusted and adjusted models yet remained consistent. Compared with phenotype 1, phenotype 4 had a non-significantly higher risk of developing asthma (17% vs. 30%; adjOR 2.28; 95% CI, 0.68–7.62; *p* = 0.18; **Figure E7**). In phenotyping with 3- and 5-class models, the alluvial plot (**Figure E8**) demonstrates a consistency of the original phenotypes (phenotypes 1–4) across the different number-class chosen. For example, in a 5-class model, phenotype A, B, C, or D had 100% concordance with the original phenotype 1, 2, 3, or 4, respectively (**Figure E9**; **Table E4**). Compared to phenotype A, phenotype E—which is concordant with phenotypes 2 and 4 (i.e., virus^RSV-low/RV^ phenotypes)—had a significantly higher asthma risk (19% vs. 60%; adjOR, 2.21; 95% CI, 1.27–3.85; *p* = 0.005; E-value = 2.34; **Figure E10**).

## Discussion

By integrating tIgE and virus data from a multicenter prospective cohort of infants hospitalized for bronchiolitis, we identified 4 clinically distinct phenotypes. For example, compared with the reference phenotype 1, infants with phenotypes 3 and 4 (tIgE^high^) had distinct biological characteristics (e.g., depleted type I interferon pathways and enriched antigen presentation pathways) based on upper airway mRNA and miRNA data. Furthermore, infants with phenotype 4 (tIgE^high^virus^RSV-high/RV^) had a significantly higher risk of developing asthma and depleted pathways related to airway epithelium structure. The sensitivity analysis revealed the robustness of our findings. To the best of our knowledge, this is the first investigation that has identified IgE-virus phenotypes of infant bronchiolitis and their longitudinal relationship with the risks of developing asthma.

Bronchiolitis has conventionally deemed a single disease with similar pathobiological mechanisms (3). However, recent research has indicated that bronchiolitis is a syndrome with high heterogeneity (4–6). Our phenotypes derived through tIgE and virus data are in agreement with studies that have evaluated the heterogeneity in terms of atopic predisposition and respiratory virus (7, 8, 16, 17). For example, although a previous single-center study has shown, in 206 infants with RSV bronchiolitis, no association between tIgE levels and developing asthma (32), our previous multicenter study has revealed, in 921 infants hospitalized for bronchiolitis, the association of a higher tIgE level with an increased risk of developing childhood asthma (17). Additionally, another analysis has found that among infants hospitalized for bronchiolitis, those with IgE-mediated sensitization (to either food or aeroallergens) and RV type C infections had increased risks of developing childhood asthma (16). Furthermore, research using three cohort data of infants with bronchiolitis in the U.S. and Finland has reported the association of profiles based on atopic predisposition (e.g., previous history of eczema and parental history of asthma) and RV infection with different risks of developing asthma (8). Likewise, recent studies using omics data have also found endotypes based on the proteome (10), metabolome (11), and microbiome (metatranscriptome) (9) data. For example, an analysis of the nasopharyngeal metabolome (11) and microbiome (9) data of infants with bronchiolitis has reported that an endotype with downregulated type I interferon pathway had a higher risk of developing asthma. The current study builds on these earlier findings and extends them by identifying distinct tIgE-virus phenotypes with differential risks of developing asthma and their biological characteristics by using upper airway mRNA and miRNA data. A better understanding of tIgE-driven phenotypes may inform potential prevention strategies (e.g., the use of monoclonal anti-IgE antibody) against asthma in young infants.

Although the exact mechanisms underlying the observed phenotypes warrant further investigation, the current study found that tIgE^high^ phenotypes 3 and 4 had depleted type I interferon pathways and enriched antigen presentation pathways. Consistently, earlier studies of children and adults with atopic predisposition have demonstrated an inverse association between tIgE levels and virus-stimulated interferon-α production in peripheral blood plasmacytoid dendritic cells (18–20). Research has also revealed that FcϵRI expression is inversely related to interferon-α production and further inhibited by IgE-mediated cross-linking of FcϵRI (19, 20). Furthermore, a previous study has demonstrated, in RV infection to bronchial epithelial cells of adults with asthma, decreased interferon-β production, impaired apoptosis, and increased virus replication (33). In addition to dysregulated type I interferon pathways, phenotypes 3 and 4 had enriched antigen presentation pathways (e.g., MHC class II). Human MHC class II is highly expressed on the surface of airway epithelial cells and antigen-presenting cells to regulate the immune system, especially in allergic diseases and viral infections (34). Consistent with these phenotypes, genome-wide studies have also reported the association of single-nucleotide polymorphisms related to human MHC class II with tIgE level (35), IgE-mediated sensitization to aeroallergens (36), and asthma (35, 37). Additionally, another study has reported increased MHC class II expression in bronchial epithelial cells of adults with asthma (38). Furthermore, previous studies have demonstrated increased MHC class II expression in pulmonary mononuclear cells of RSV-infected mice (39), not in RV-infected alveolar epithelial cell lines (40).

Of the tIgE^high^ phenotypes, phenotype 4 (tIgE^high^virus^RSV-low/RV^), which is at a higher risk of asthma, had depleted airway epithelial structure (e.g., cilia and intercellular junction) pathways. Consistent with our observations, growing evidence also suggests that airway epithelial abnormality and dysfunction were observed in adults and children with asthma (41, 42). For example, ciliated epithelium in the airway serves as a physical barrier by facilitating the clearance of mucus (43). Previous research using bronchial epithelial cells of adults (44) and children (45) with asthma has shown ciliary ultrastructural abnormality (e.g., loss of ciliated cells and ciliary disorientation) and dysfunction (e.g., low beat frequency). Likewise, intercellular junctions (e.g., tight junctions) also serve as an airway epithelial barrier by regulating permeability (46). In adults with asthma, tight junctions are disrupted, and epithelial permeability in bronchial epithelial cells is increased (47). Additionally, in airway epithelial cells of children with asthma, RV infection reduces the expression of tight junctional protein and increases permeability (48). Regardless of the complexity of these potential mechanisms, the current study has revealed that tIgE- and virus-derived phenotypes have distinct biological characteristics and differential asthma risks. Our data should advance research into developing strategies for asthma prevention based on tIgE-virus phenotyping.

The current study has several potential limitations. First, the current study did not have non-bronchiolitis “controls”. Nevertheless, this study did not aim to elicit infant phenotypes related to the incidence of bronchiolitis but to examine bronchiolitis phenotypes and their risk of developing asthma. Second, while the analytic and non-analytic cohorts did not differ in most clinical characteristics, the proportion of RSV infection was significantly different. Therefore, the phenotypes in the analytic cohort may not completely represent those in MARC-35. Third, although bronchiolitis involves inflammation in both upper and lower airways, the current study relied on upper airway mRNA and miRNA data. Nonetheless, research has shown that upper airway data represent inflammatory profiles in the lower airway, which is more invasive and difficult to collect in young infants (49). Fourth, the mRNA and miRNA data were derived from different upper airway specimens. Additionally, these specimens comprised heterogeneous cell populations, limiting pathways in the upper airway among the phenotypes. Fifth, these mRNA and miRNA data were measured only at the timing of bronchiolitis hospitalization. Although longitudinal measurement would be helpful, the single measurement successfully characterized the biologically distinct phenotypes. Sixth, in the analysis using the mRNA and miRNA data, the threshold of statistical significance was set at FDR <0.10. However, in the mRNA data, most identified pathways met FDR of <0.05. Seventh, in the sensitivity analysis excluding infants with a previous history of breathing problems, the phenotype-outcome associations did not show a statistical significance. However, the associations were directionally consistent between the main and sensitivity analysis. The consistency could support the robustness of the main analysis. Eighth, the asthma diagnosis may have been misclassified, and some children are going to develop asthma later. To address the potential limitations, the cohort is currently being followed until age 9 years. Ninth, while our findings are biologically and clinically plausible, they warrant further validation in an independent cohort. Lastly, our inferences may not be generalizable to populations other than infants hospitalized for bronchiolitis. Nevertheless, our data are highly relevant to >110,000 infants hospitalized for bronchiolitis annually in the U.S.—a population with a substantial morbidity burden.

## Conclusions

By applying an unsupervised clustering approach to the tIgE and virus data from a prospective multicenter cohort study of infants hospitalized for bronchiolitis, we identified 4 clinically meaningful phenotypes. Specifically, the phenotype characterized by a high tIgE level and RSV (with a low genomic load) and RV infection had the greatest risk of developing asthma. Additionally, by using upper airway mRNA and miRNA data, these phenotypes revealed biologically distinct airway responses (e.g., type I interferon, antigen presentation, and airway epithelium structure pathways) during bronchiolitis. These findings offer an evidence-base for the early identification of high-risk infants during a critical period of airway development. Furthermore, these findings should advance asthma prevention strategies (e.g., modulating immune response by biologics targeting IgE, such as omalizumab) in this large patient population with a high morbidity burden.

## Data availability statement

The data that support the findings of this study are available on request from the corresponding author through controlled access to be compliant with the informed consent forms of the MARC studies. The data are not publicly available due to privacy and ethical restrictions.

## Ethics statement

The studies involving human participants were reviewed and approved by Massachusetts General Hospital. Written informed consent to participate in this study was provided by the participants’ legal guardian/next of kin.

## Author contributions

Dr. RS carried out the main statistical analysis, drafted the initial manuscript, and approved the final manuscript as submitted. Drs. ZZ and TO reviewed and revised the initial manuscript, and approved the final manuscript as submitted. Drs. RF, JM, and ST collected the study data, reviewed and revised the initial manuscript, and approved the final manuscript as submitted. Drs. IR-T and MP-L carried out the bioinformatic analyses of the genomic data, reviewed and revised the initial manuscript, and approved the final manuscript as submitted. Drs. KH and CC conceptualized the study, obtained funding, supervised the statistical analysis, reviewed and revised the initial manuscript, and approved the final manuscript as submitted. All authors contributed to the article and approved the submitted version.
