# Supplementary material for: Immunoglobulin E-virus phenotypes of infant bronchiolitis and risk of childhood asthma
Source: Front Immunol. 2023 May 10;14:1187065. doi: 10.3389/fimmu.2023.1187065 (PMC10205992; doi:10.3389/fimmu.2023.1187065)
Supplement: Supplementary file 1 [file DataSheet_1.docx]

SUPPLEMENTARY MATERIAL

Table of Contents:

Supplementary Methods 3

Study design, setting, and participants 3

Data collection 3

Nasopharyngeal total RNA extraction, RNA-seq, quality control, and mRNA profiling 4

Nasal total RNA extraction, small RNA-seq, quality control, and microRNA profiling 5

Outcome measure 5

Statistical analysis 6

Supplementary References 9

Table E1. Principal investigators at the 17 participating sites in MARC-35 13

Table E2. Baseline patient characteristics and clinical course of infants hospitalized for bronchiolitis in MARC-35, according to 4 phenotypes 14

Table E3. Comparisons between analytic and non-analytic cohorts in MARC-35 17

Table E4. Baseline patient characteristics and clinical course of infants hospitalized for bronchiolitis in the analytic cohort, using for 5 phenotypes in the sensitivity analysis 19

Figure E1. Consensus matrices and cumulative distribution function plot to identify an optimal number of the virus clusters 22

Figure E2. Consensus matrices and cumulative distribution function plot to identify an optimal number of the total immunoglobulin E-virus clusters 23

Figure E3. Differential gene expression analysis between phenotypes in infants hospitalized for bronchiolitis 24

Figure E4. Between-phenotype difference (1 vs. 2) in nasopharyngeal mRNA pathways among infants hospitalized for bronchiolitis 25

Figure E5. Differential microRNA expression analysis between phenotypes in infants hospitalized for bronchiolitis 26

Figure E6. Between-phenotype difference (1 vs. 2) in nasopharyngeal mRNA pathways and nasal microRNA pathways among infants hospitalized for bronchiolitis 27

Figure E7. Association of phenotypes of infant bronchiolitis with risk of developing childhood asthma, limiting to infants without a previous history of breathing problems in the sensitivity analysis. 28

Figure E8. Alluvial plot to examine consistencies across different numbers of phenotypes 29

Figure E9. Clinical and virus characteristics of infants hospitalized for bronchiolitis, according to phenotypes, by using for 5 phenotypes in the sensitivity analysis 30

Figure E10. Association of phenotypes of infant bronchiolitis with risk of developing childhood asthma, using 5 phenotypes in the sensitivity analysis 31

# Supplementary Methods

## Study design, setting, and participants

We analyzed data from an ongoing, multicenter, prospective cohort study of infants (age < 1 year) hospitalized with bronchiolitis. This study, called the 35th Multicenter Airway Research Collaboration (MARC-35),^1–3^ is coordinated by the Emergency Medicine Network (EMNet),^4^ a research collaboration of 247 participating hospitals. Using a standardized protocol,^1–3^ the investigators at 17 sites across 14 U.S. states (**Table E1**) enrolled infants hospitalized with an attending physician diagnosis of bronchiolitis during one of the consecutive bronchiolitis seasons from November 1 to April 30 (2011–2014). Bronchiolitis was defined by the American Academy of Pediatrics (AAP) guidelines—acute respiratory illness with some combination of rhinitis, cough, tachypnea, wheezing, crackles, and retractions, regardless of previous breathing problem episodes.^5^ We excluded infants with a known heart-lung disease, immunodeficiency, immunosuppression, or gestational age < 32 weeks. All patients were treated at the discretion of the treating physician. The institutional review board at each participating hospital approved the study. Written informed consent was obtained from the parent or guardian.

## Data collection

Investigators conducted a structured interview that assessed patients’ demographic characteristics, medical, environmental, and family history, and details of the bronchiolitis course. After the index hospitalization for bronchiolitis, we conducted parental interviews by telephone at 6-month intervals and reviewed medical records (only by trained physicians). All data were reviewed at the EMNet Coordinating Center at Massachusetts General Hospital (Boston, MA, U.S.), and site investigators were queried about missing data and discrepancies identified by manual data checks.

Using standard protocols,^6,7^ serum, nasopharyngeal, and nasal specimens were collected by trained site investigators within 24 hours of hospitalization. Serum total immunoglobulin E (tIgE) was measured by using ImmunoCAP Total IgE (Thermo Fisher Scientific, Waltham, MA, U.S.); the range of this assay is 2 to 5,000 kU/L. Specific immunoglobulin E (IgE) was measured at enrollment by using ImmunoCAP Specific IgE (Thermo Fisher Scientific). A positive test result was defined as 0.35 kU/L. IgE sensitization was defined by having one or more positive values for serum aeroallergen or food allergen-specific IgE. Nasopharyngeal specimens were tested for respiratory viruses (e.g., respiratory syncytial virus [RSV] and rhinovirus [RV]) using real-time polymerase chain reaction assays at the Baylor College of Medicine (Houston, TX, Massachusetts)^6^ and mRNA profiling by RNA sequencing (RNA-seq). Nasal specimens were tested for miRNA profiling by small RNA-seq.

## Nasopharyngeal total RNA extraction, RNA-seq, quality control, and mRNA profiling

Total RNA was isolated from 244 randomly-selected nasopharyngeal specimens using Trizol LS reagent (ThermoFisher Scientific) in combination with the Direct-zol RNA Miniprep Kit (Zymo Research, Irvine, CA, U.S.). RNA quantity was measured with the Qubit 2.0 fluorometer (ThermoFisher Scientific). Its quality was assessed with the Agilent Bioanalyzer 2100 (Agilent, Palo Alto, CA, U.S.) using the RNA 6000 Nano kit, and no detection of DNA contamination was confirmed based on the Bioanalyzer results. Total RNA underwent DNase treatment using the TURBO 21 DNA-free™ Kit (ThermoFisher Scientific) for the removal of DNA contamination and underwent rRNA reduction for both human and bacterial rRNA using NEBNext rRNA Depletion Kits (New England Biolabs, Ipswich, MA, U.S.). RNA was prepared for sequencing using the NEBNext Ultra II Directional RNA Library Prep Kit (New England Biolabs) and sequenced on an NovaSeq6000 (Illumina, San Diego, CA, U.S.) using an S4 100 bp PE Flowcell (Illumina). Raw sequence reads were trimmed and filtered for adapters and contaminants using the k-mers strategy in bbduk^8^ and default settings (i.e., minlength=14, minavgquality=20, maxns=0). All RNA-seq samples had sufficient sequence depth (mean of 8,067,019 pair-end reads/sample) to obtain a high degree of sequence coverage.

We estimated transcript abundances from clean RNA-seq reads by Salmon^9^ using the human transcriptome (hg38) and the mapping-based mode. We first generated a decoy-aware transcriptome and then quantified the reads using Salmon’s default settings and the following flags: –validateMappings, –recoverOrphans, –seqBias, and –gcBias. Salmon corrects for potential changes in gene length across specimens (e.g., from differential isoform usage) and has higher sensitivity at the same false discovery rate (FDR) in differential expression gene analysis. Next, by calculating log_10_-transformed total read count per specimen of the 244 mRNA data, we filtered the 10 mRNA data with the low read count defined as less than the mean minus 2.5 standard deviations (SD). Lastly, we normalize the read count by the R *DESeq2* package^10^ using default settings.

## Nasal total RNA extraction, small RNA-seq, quality control, and microRNA profiling

Total RNA was isolated from nasal swab specimens and the quality and quantity were assessed using the same protocol as the nasopharyngeal specimens. RNA in 624 speciemens with sufficient quantity and quality was prepared for sequencing using PerkinElmer NEXTFLEX® small RNA-seq v3 kit with Unique Dual Indexes (PerkinElmer, Waltham, MA, U.S.) and sequenced on an Illumina NovaSeq6000 using an S2 50 bp PE Flowcell (Illumina). All small RNA-seq samples had sufficient sequence depth (mean of 26,736,490 pair-end reads/sample) to obtain a high degree of sequence coverage.

From clean small RNA-seq reads, we estimated microRNA (miRNA) detection and abundance by sMETASeq.^11^ Fastq files underwent quality control in cutadapt^12^ and were collapsed into unique reads. We mapped trimmed reads against human miRNA sequences from miRBase V22.^13^ Next, by calculating log_10_-transformed total read count per specimen of the 624 miRNA data, we removed the 24 miRNA data with the low read count defined as less than the mean minus 2.5 SD. Lastly, we normalized the read count by the R *DESeq2* package^10^ using default settings.

## Outcome measure

The outcome of interest was the development of asthma by age 6 years. Asthma was defined using a commonly used epidemiologic definition^14,15^: physician diagnosis of asthma, with either asthma medication use (e.g., inhaled bronchodilators and inhaled corticosteroids) or asthma-related symptoms (e.g., wheezing and nocturnal cough) during the year before the evaluation at age 6 years.

## Statistical analysis

The objectives of the current study are 1) to identify tIgE-virus phenotypes among infants hospitalized for bronchiolitis, 2) to examine their relationships with the risk of developing asthma, and 3) to determine their biological characteristics with the use of the mRNA and miRNA data. The analytic workflow is summarized in **Figure 1**.

First, we identified mutually exclusive clusters for each of the tIgE and virus datasets of MARC-35 infants collected at the index hospitalization. For the tIgE data, we generated the low and high tIgE clusters by using the median value. In contrast to tIgE clustering with the use of median cut-off value, for the virus data, we used RSV (binary), RV species A and C (binary), and their genomic load (count) represented by cycle threshold (Ct) values based on their importance in bronchiolitis and asthma.^16^ When the virus was not detected, we imputed the Ct values with 40. Then, we computed a Gower distance using the R *StatMatch* package^17^ and derived the virus clusters by using a consensus clustering algorithm with partitioning around medoids (PAM) method using the R *ConsensusClusterPlus* package.^18,19^ To choose an optimal number of the virus clusters, we used a combination of separations of the consensus matrix and relative change of the area under the cumulative distribution function (CDF) curve, in addition to the cluster size and clinical plausibility.^6,20–23^ Second, we combined the tIgE and virus clusters to derive a fused matrix, computed a Gower distance, and identified mutually exclusive 4 phenotypes by using a consensus clustering algorithm with PAM method. Third, to interpret the clinical characteristics of the phenotypes**,** we developed chord diagrams on the relationship of the phenotypes with major clinical and virus variables using the R *circlize* package.^24^

Fourth, we determined the longitudinal association of the phenotypes with the asthma risk. Of the 1,016 MARC-35 infants, 234 and 600 infants had the nasopharyngeal mRNA and nasal miRNA data with a read count with >the mean minus 2.5 SD, respectively. Of the 187 infants with both data, we used 182 infants with the asthma outcome data as the analytic cohort. We first examined the differences between the analytic and non-analytic cohorts in the patient characteristics, clinical presentation, and detected respiratory viruses by using the chi-square and Wilcoxon rank sum tests as appropriate. Additionally, between the 4 phenotypes in the analytic cohort, we compared the baseline clinical characteristics by using the chi-square and Kruskal-Wallis tests as appropriate. Then, for the analytic cohort, we constructed unadjusted logistic regression and multivariable mixed-effects logistic regression models. In the multivariable mixed-effects model, we adjusted for site effects and patient-level potential confounders (i.e., age, sex, parental history of asthma, prematurity [<37 weeks], pre-hospitalization use of inhaled and/or systemic corticosteroids). We chose these covariates based on clinical plausibility and *a priori* knowledge.^25,26^

Fifth, we examined the difference in biological characteristics between the derived phenotypes by using the nasopharyngeal mRNA data. We first conducted differential expression gene analysis between the phenotypes by using the R *DESeq2* package.^10^ Next, by using the rank gene list with Wald statistic (i.e., log_2_ fold change divided by standard error) in the differential expression gene analysis, we conducted Gene Set Enrichment Analysis (GSEA)^27,28^ based on Biological Processes in Gene Ontology by the R *clusterProfiler* package (FDR <.10).^29,30^

Sixth, we also examined the difference in biological characteristics by integrating the nasal miRNA data with the nasopharyngeal mRNA data. As with the mRNA data, we conducted differential expression miRNA analysis by using the R *DESeq2* package^10^ and miRNA set enrichment analysis by the rank miRNA list based on Biological Processes in Gene Ontology derived from the miRNA-target databases, miRTarBase 8.0,^31^ using miEAA 2.0^32^ via the R *rbioapi* patckage.^33^ We identified pathways that are enriched in the mRNA data and depleted in the miRNA data or that are depleted in the mRNA data and enriched in the miRNA data (FDR <.10).

In the sensitivity analysis, we first computed E-values to determine the robustness of causal inference to potential unmeasured confounding by using the R *EValue* package.^34^ The E-value represents the minimum magnitude of association that a set of unmeasured confounders would need to have in order to fully explain the association of interest, conditional on the covariates. For example, an E-value of 2.0 means that the odds ratio for the association of unmeasured confounders with both the exposure and outcome would have to be ≥2.0 to explain away the observed exposure-outcome association. We next examined the phenotype-outcome associations after excluding infants with a previous history of breathing problem. We also examined the robustness of the phenotype-outcome associations under different definitions of exposure^35^ by repeating the analysis using a different number of phenotypes.

Analysis used R version 4.1.2 (R Foundation, Vienna, Austria). All *P* values were two-tailed, with *P* <.05 considered statistically significant. We computed the Benjamini-Hochberg FDR that allows for the interpretation of statistical significance in the context of multiple hypothesis testing,^36^ with FDR <.10 considered statistically significant.

# Supplementary References

1. Stewart CJ, Mansbach JM, Wong MC, Ajami NJ, Petrosino JF, Camargo CA Jr, et al. Associations of Nasopharyngeal Metabolome and Microbiome with Severity among Infants with Bronchiolitis. A Multiomic Analysis. Am J Respir Crit Care Med 2017;196:882–91.

2. Hasegawa K, Mansbach JM, Ajami NJ, Espinola JA, Henke DM, Petrosino JF, et al. Association of nasopharyngeal microbiota profiles with bronchiolitis severity in infants hospitalised for bronchiolitis. Eur Respir J 2016;48:1329–39.

3. Hasegawa K, Mansbach JM, Ajami NJ, Petrosino JF, Freishtat RJ, Teach SJ, et al. Serum cathelicidin, nasopharyngeal microbiota, and disease severity among infants hospitalized with bronchiolitis. J Allergy Clin Immunol 2017;139:1383–86.e6.

4. Emergency Medicine Network. Available from: https://www.emnet-usa.org/

5. Ralston SL, Lieberthal AS, Meissner HC. Ralston SL, Lieberthal AS, Meissner HC, et al. Clinical Practice Guideline: The Diagnosis, Management, and Prevention of Bronchiolitis. Pediatrics. 2014;134(5):e1474–502.

6. Hasegawa K, Mansbach JM, Bochkov YA, Gern JE, Piedra PA, Bauer CS, et al. Association of Rhinovirus C Bronchiolitis and Immunoglobulin E Sensitization During Infancy With Development of Recurrent Wheeze. JAMA Pediatr 2019;173:544–52.

7. Stewart CJ, Mansbach JM, Ajami NJ, Petrosino JF, Zhu Z, Liang L, et al. Serum Metabolome Is Associated With the Nasopharyngeal Microbiota and Disease Severity Among Infants With Bronchiolitis. J Infect Dis 2019;219:2005–14.

8. Bushnell B. BBMap: A Fast, Accurate, Splice-Aware Aligner. Available at: <https://www.osti.gov/servlets/purl/1241166>. Accessed December 26, 2022.

9. Patro R, Duggal G, Love MI, Irizarry RA, Kingsford C. Salmon provides fast and bias-aware quantification of transcript expression. Nat Methods 2017;14:417–9.

10. Love MI, Huber W, Anders S. Moderated estimation of fold change and dispersion for RNA-seq data with DESeq2. Genome Biol 2014;15:550.

11. Mjelle R, Aass KR, Sjursen W, Hofsli E, Sætrom P. sMETASeq: Combined Profiling of Microbiota and Host Small RNAs. iScience 2020;23:101131.

12. Martin M. Cutadapt removes adapter sequences from high-throughput sequencing reads. EMBnet.journal 2011;17:10–2.

13. Kozomara A, Birgaoanu M, Griffiths-Jones S. miRBase: from microRNA sequences to function. Nucleic Acids Res 2019;47:D155–62.

14. Camargo CA Jr, Ingham T, Wickens K, Thadhani R, Silvers KM, Epton MJ, et al. Cord-blood 25-hydroxyvitamin D levels and risk of respiratory infection, wheezing, and asthma. Pediatrics 2011;127:e180-7.

15. Nanishi M, Fujiogi M, Stevenson M, Liang L, Qi YS, Raita Y, et al. Association of Growth Trajectory Profiles with Asthma Development in Infants Hospitalized with Bronchiolitis. J Allergy Clin Immunol Pract 2022;10:723-731.e5.

16. Hasegawa K, Dumas O, Hartert TV, Camargo CA Jr. Advancing our understanding of infant bronchiolitis through phenotyping and endotyping: clinical and molecular approaches. Expert Rev Respir Med 2016;10:891–9.

17. D’Orazio M. Integration and imputation of survey data in R: the StatMatch package. *Romanian Statistical Review* 2015;63(2):57-68.

18. Wilkerson MD, Hayes DN. ConsensusClusterPlus: a class discovery tool with confidence assessments and item tracking. Bioinformatics 2010;26:1572–3.

19. Monti S, Tamayo P, Mesirov J, Golub T. Consensus Clustering: A Resampling-Based Method for Class Discovery and Visualization of Gene Expression Microarray Data. Mach Learn 2003;52:91–118.

20. Raita Y, Pérez-Losada M, Freishtat RJ, Harmon B, Mansbach JM, Piedra PA, et al. Integrated omics endotyping of infants with respiratory syncytial virus bronchiolitis and risk of childhood asthma. Nat Commun 2021;12:3601.

21. Raita Y, Camargo CA Jr, Bochkov YA, Celedón JC, Gern JE, Mansbach JM, et al. Integrated-omics endotyping of infants with rhinovirus bronchiolitis and risk of childhood asthma. J Allergy Clin Immunol 2021;147:2108–17.

22. Ooka T, Raita Y, Fujiogi M, Freishtat RJ, Gerszten RE, Mansbach JM, et al. Proteomics endotyping of infants with severe bronchiolitis and risk of childhood asthma. Allergy 2022;77:3350–61.

23. Fujiogi M, Zhu Z, Raita Y, Ooka T, Celedon JC, Freishtat R, et al. Nasopharyngeal lipidomic endotypes of infants with bronchiolitis and risk of childhood asthma: a multicentre prospective study. Thorax 2022;77:1059–69.

24. Gu Z, Gu L, Eils R, Schlesner M, Brors B. circlize Implements and enhances circular visualization in R. Bioinformatics 2014;30:2811–2.

25. Hasegawa K, Jartti T, Mansbach JM, Laham FR, Jewell AM, Espinola JA, et al. Respiratory syncytial virus genomic load and disease severity among children hospitalized with bronchiolitis: multicenter cohort studies in the United States and Finland. J Infect Dis 2015;211:1550–9.

26. Mansbach JM, Piedra PA, Teach SJ, Sullivan AF, Forgey T, Clark S, et al. Prospective multicenter study of viral etiology and hospital length of stay in children with severe bronchiolitis. Arch Pediatr Adolesc Med 2012;166:700–6.

27. Subramanian A, Tamayo P, Mootha VK, Mukherjee S, Ebert BL, Gillette MA, et al. Gene set enrichment analysis: a knowledge-based approach for interpreting genome-wide expression profiles. Proc Natl Acad Sci U S A 2005;102:15545–50.

28. Mootha VK, Lindgren CM, Eriksson KF, Subramanian A, Sihag S, Lehar J, et al. PGC-1alpha-responsive genes involved in oxidative phosphorylation are coordinately downregulated in human diabetes. Nat Genet 2003;34:267–73.

29. Yu G, Wang LG, Han Y, He QY. clusterProfiler: an R package for comparing biological themes among gene clusters. OMICS 2012;16:284–7.

30. Wu T, Hu E, Xu S, Chen M, Guo P, Dai Z, et al. clusterProfiler 4.0: A universal enrichment tool for interpreting omics data. Innovation (Camb) 2021;2:100141.

31. Huang HY, Lin YCD, Li J, Huang KY, Shrestha S, Hong HC, et al. miRTarBase 2020: updates to the experimentally validated microRNA-target interaction database. Nucleic Acids Res 2020;48:D148–54.

32. Kern F, Fehlmann T, Solomon J, Schwed L, Grammes N, Backes C, et al. miEAA 2.0: integrating multi-species microRNA enrichment analysis and workflow management systems. Nucleic Acids Res 2020;48:W521–8.

33. Rezwani M, Pourfathollah AA, Noorbakhsh F. rbioapi: user-friendly R interface to biologic web services’ API. Bioinformatics 2022;38:2952–3.

34. Package ‘EValue’ sensitivity analyses for unmeasured confounding and other biases in observational studies and meta-analyses. Available at: <https://cran.r-project.org/web/packages/EValue/EValue.pdf> Accessed December 26, 2022.

35. Thabane L, Mbuagbaw L, Zhang S, Samaan Z, Marcucci M, Ye C, Thabane M, Giangregorio L, Dennis B, Kosa D, et al. A tutorial on sensitivity analyses in clinical trials: the what, why, when and how. BMC Med Res Methodol (2013) 13:92.

36. Benjamini Y, Hochberg Y. Controlling the false discovery rate: A practical and powerful approach to multiple testing. J R Stat Soc 1995;57:289–300.

# Table E1. Principal investigators at the 17 participating sites in MARC-35

| Amy D. Thompson, MD | Alfred I. duPont Hospital for Children, Wilmington, DE |
| --- | --- |
| Federico R. Laham, MD, MS | Arnold Palmer Hospital for Children, Orlando, FL |
| Jonathan M. Mansbach, MD, MPH | Boston Children’s Hospital, Boston, MA |
| Vincent J. Wang, MD, MHA and Susan Wu, MD | Children’s Hospital of Los Angeles, Los Angeles, CA |
| Michelle B. Dunn, MD and Jonathan M. Spergel, MD, PhD | Children’s Hospital of Philadelphia, Philadelphia, PA |
| Juan C. Celedón, MD, DrPH | Children’s Hospital of Pittsburgh, Pittsburgh, PA |
| Michael R. Gomez, MD, MS-HCA and Nancy Inhofe, MD | The Children’s Hospital at St. Francis, Tulsa, OK |
| Brian M. Pate, MD and Henry T. Puls, MD | The Children’s Mercy Hospital & Clinics, Kansas City, MO |
| Stephen J. Teach, MD, MPH | Children’s National Medical Center, Washington, D.C. |
| Richard T. Strait, MD and Stephen C. Porter, MD, MSc, MPH | Cincinnati Children’s Hospital and Medical Center, Cincinnati, OH |
| Ilana Y. Waynik, MD | Connecticut Children’s Medical Center, Hartford, CT |
| Sujit Iyer, MD | Dell Children’s Medical Center of Central Texas, Austin, TX |
| Michelle D. Stevenson, MD, MS | Norton Children’s Hospital, Louisville, KY |
| Margaret Samuels-Kalow, MD, MPhil, Wayne G. Shreffler, MD, PhD, Ari R. Cohen, MD | Massachusetts General Hospital, Boston, MA |
| Anne K. Beasley, MD and Cindy S. Bauer,  MD | Phoenix Children’s Hospital, Phoenix, AZ |
| Thida Ong, MD and Markus Boos, MD,  PhD | Seattle Children’s Hospital, Seattle, WA |
| Charles G. Macias, MD, MPH | Texas Children's Hospital, Houston, TX |

# Table E2. Baseline patient characteristics and clinical course of infants hospitalized for bronchiolitis in MARC-35, according to 4 phenotypes

| **Variables** | **Overall**  **(n = 1,016; 100%)** | **Phenotype 1**  **(tIgE^low^**  **virus^RSV-high^)**  **(n = 232; 23%)** | **Phenotype 2**  **(tIgE^low^**  **Virus^RSV-low/RV^)**  **(n = 276; 27%)** | **Phenotype 3**  **(tIgE^high^**  **virus^RSV-high^)**  **(n = 225; 22%)** | **Phenotype 4**  **(tIgE^high^**  **virus^RSV-low/RV^)**  **(n = 283; 28%)** | ***P* value^†^** |
| --- | --- | --- | --- | --- | --- | --- |
| Demographics |  |  |  |  |  |  |
| Age (month), median (IQR) | 3 (2–6) | 2 (1–3) | 2 (1–4) | 4 (2–7) | 5 (3–8) | <.001^‡^ |
| Male sex | 610 (60) | 132 (57) | 163 (59) | 134 (60) | 181 (64) | .41 |
| Race/ethnicity |  |  |  |  |  | <.001 |
| Non-Hispanic white | 430 (42) | 116 (50) | 148 (54) | 87 (39) | 79 (28) |  |
| Non-Hispanic black | 239 (24) | 47 (20) | 52 (19) | 50 (22) | 90 (32) |  |
| Hispanic | 308 (30) | 61 (26) | 66 (24) | 75 (33) | 106 (37) |  |
| Other | 39 (4) | 8 (3) | 10 (4) | 13 (6) | 8 (3) |  |
| C-section delivery | 348 (34) | 83 (36) | 85 (31) | 86 (38) | 94 (33) | .34 |
| Prematurity (32–36.9 weeks) | 186 (18) | 44 (19) | 55 (20) | 39 (17) | 48 (17) | .79 |
| History of eczema | 149 (15) | 21 (9) | 19 (7) | 45 (20) | 64 (23) | <.001 |
| Previous breathing problems (count) |  |  |  |  |  | <.001 |
| 0 | 810 (80) | 209 (90) | 230 (83) | 190 (84) | 181 (64) |  |
| 1 | 160 (16) | 21 (9) | 34 (12) | 24 (11) | 81 (29) |  |
| ≥2 | 46 (5) | 2 (1) | 12 (4) | 11 (5) | 21 (7) |  |
| Lifetime corticosteroid use^§^ | 147 (14) | 23 (10) | 34 (12) | 30 (13) | 60 (21) | .001 |
| Pre-hospitalization corticosteroid use^¶^ | 98 (10) | 15 (6) | 27 (10) | 19 (8) | 37 (13) | .076 |
| Ever attended daycare | 234 (23) | 32 (14) | 55 (20) | 65 (29) | 82 (29) | <.001 |
| Cigarette smoke exposure at home | 156 (15) | 35 (15) | 35 (13) | 34 (15) | 52 (18) | .32 |
| Parental history of eczema | 198 (19) | 35 (15) | 52 (19) | 48 (21) | 63 (22) | .19 |
| Parental history of asthma | 345 (34) | 65 (28) | 90 (33) | 86 (38) | 104 (37) | .083 |
| Clinical presentation at index hospitalization |  |  |  |  |  |  |
| Weight (kg), median (IQR) | 6.1 (4.7–7.7) | 5.1 (4.2–6.2) | 5.3 (4.3–6.8) | 6.9 (5.5–8.4) | 7.3 (5.8–8.6) | <.001^‡^ |
| Respiratory rate (per minute), median (IQR) | 48 (40–60) | 50 (40–60) | 48 (40–60) | 48 (40–60) | 49 (40–60) | .18^‡^ |
| Oxygen saturation |  |  |  |  |  | .59 |
| <90% | 91 (9) | 25 (11) | 24 (9) | 15 (7) | 27 (10) |  |
| 90–93.9% | 155 (15) | 32 (14) | 36 (13) | 43 (19) | 44 (16) |  |
| ≥94% | 749 (74) | 169 (73) | 211 (76) | 162 (72) | 207 (73) |  |
| Blood testing |  |  |  |  |  |  |
| Blood eosinophilia (≥4%) | 96 (9) | 25 (11) | 34 (12) | 13 (6) | 24 (8) | .10 |
| tIgE (kU/L), median (IQR) | 4.4 (1.9–12.5) | 1.9 (1.9–2.6) | 1.9 (1.9–2.5) | 11.1 (6.6–22.3) | 13.7 (7.2–29.9) | <.001^‡^ |
| Allergic (specific IgE) sensitization | 204 (20) | 18 (8) | 14 (5) | 74 (33) | 98 (35) | <.001 |
| Aeroallergen sensitization | 16 (2) | 2 (1) | 1 (0) | 8 (4) | 5 (2) | .027 |
| Food sensitization | 193 (19) | 16 (7) | 13 (5) | 68 (30) | 96 (34) | <.001 |
| Viral testing |  |  |  |  |  |  |
| RSV | 821 (81) | 232 (100) | 195 (71) | 225 (100) | 169 (60) | <.001 |
| RSV cycle threshold values | 22 (20–25) | 21 (19–22) | 26 (24–28) | 21 (20–22) | 25 (24–28) | <.001^‡^ |
| RV | 214 (21) | 1 (0) | 106 (38) | 4 (2) | 103 (36) | <.001 |
| Other pathogens**^††^** | 237 (23) | 25 (11) | 64 (23) | 41 (18) | 107 (38) | <.001 |
| Clinical course |  |  |  |  |  |  |
| Positive pressure ventilation use^‡‡^ | 55 (5) | 22 (9) | 12 (4) | 8 (4) | 13 (5) | .019 |
| Intensive treatment use^§§^ | 163 (16) | 42 (18) | 46 (17) | 30 (13) | 45 (16) | .56 |

Note: Data are the number (percentage) of children unless otherwise indicated. Percentages may not equal 100 because of rounding and missingness. All data are collected, unless otherwise indicated.

Abbreviations: IgE, immunoglobulin E; IQR, interquartile range; RSV, respiratory syncytial virus; RV, rhinovirus; tIgE, total immunoglobulin E.

**^†^** Tested by the chi-square test, unless otherwise indicated.

^‡^ Tested by the Kruskal-Wallis test.

^§^ Defined as the use of inhaled and/or systemic corticosteroids before the index hospitalization.

^¶^ Defined as the use of inhaled and/or systemic corticosteroids for breathing problems that caused the index hospitalization.

**^††^** Adenovirus, bocavirus, *Bordetella pertussis*, enterovirus, human coronavirus NL63, OC43, 229E, or HKU1, human metapneumovirus, influenza A or B virus, *Mycoplasma pneumoniae*, and parainfluenza virus 1–3.

^‡‡^ Defined as the use of invasive and/or non-invasive mechanical ventilation (e.g., continuous positive airway pressure ventilation) during the index hospitalization.

^§§^ Defined as the use of positive pressure ventilation and/or admission to intensive care unit.

# Table E3. Comparisons between analytic and non-analytic cohorts in MARC-35

| **Variables** | **MARC-35**  **(n = 1,016)** | **Analytic**  **Cohort**  **(n = 182; 18%)** | **Non-analytic**  **cohort**  **(n = 834; 82%)** | ***P* value^†^** |
| --- | --- | --- | --- | --- |
| Demographics |  |  |  |  |
| Age (month), median (IQR) | 3 (2–6) | 3 (2–6) | 3 (2–6) | .95^‡^ |
| Male sex | 610 (60) | 104 (57) | 506 (61) | .43 |
| Race/ethnicity |  |  |  | .94 |
| Non-Hispanic white | 430 (42) | 75 (41) | 355 (43) |  |
| Non-Hispanic black | 239 (24) | 45 (25) | 194 (23) |  |
| Hispanic | 308 (30) | 56 (31) | 252 (30) |  |
| Other | 39 (4) | 6 (3) | 33 (4) |  |
| C-section delivery | 348 (34) | 62 (34) | 286 (34) | 1.00 |
| Prematurity (32–36.9 weeks) | 186 (18) | 31 (17) | 155 (19) | .70 |
| History of eczema | 149 (15) | 24 (13) | 125 (15) | .61 |
| Previous breathing problems (count) |  |  |  | .12 |
| 0 | 810 (80) | 155 (85) | 655 (79) |  |
| 1 | 160 (16) | 20 (11) | 140 (17) |  |
| ≥2 | 46 (5) | 7 (4) | 39 (5) |  |
| Lifetime corticosteroid use^§^ | 147 (14) | 31 (17) | 116 (14) | .33 |
| Pre-hospitalization corticosteroid use^¶^ | 98 (10) | 19 (10) | 79 (9) | .79 |
| Ever attended daycare | 234 (23) | 54 (30) | 180 (22) | .024 |
| Cigarette smoke exposure at home | 156 (15) | 27 (15) | 129 (15) | .92 |
| Parental history of eczema | 198 (19) | 36 (20) | 162 (19) | .99 |
| Parental history of asthma | 345 (34) | 64 (35) | 281 (34) | .77 |
| Clinical presentation at index hospitalization |  |  |  |  |
| Weight (kg), median (IQR) | 6.1 (4.7–7.7) | 6.2 (4.6–8.0) | 6.1 (4.7–7.7) | .92^‡^ |
| Respiratory rate (per minute), median (IQR) | 48 (40–60) | 48 (40–60) | 48.5 (40–60) | .40^‡^ |
| Oxygen saturation |  |  |  | .031 |
| <90% | 91 (9) | 11 (6) | 80 (10) |  |
| 90–93.9% | 155 (15) | 20 (11) | 135 (16) |  |
| ≥94% | 749 (74) | 148 (81) | 601 (72) |  |
| Blood testing |  |  |  |  |
| Blood eosinophilia (≥4%) | 96 (9) | 15 (8) | 81 (10) | .73 |
| tIgE (kU/L), median (IQR) | 4.4 (1.9–12.5) | 4.8 (1.9–15.5) | 4.3 (1.9–12.1) | .33^‡^ |
| Allergic (specific IgE) sensitization | 204 (20) | 45 (25) | 159 (19) | .10 |
| Aeroallergen sensitization | 16 (2) | 1 (1) | 15 (2) | .37 |
| Food sensitization | 193 (19) | 44 (24) | 149 (18) | .063 |
| Viral testing |  |  |  |  |
| RSV | 821 (81) | 167 (92) | 654 (78) | <.001 |
| RSV cycle threshold values | 22 (20–25) | 22 (20–25) | 22 (20–25) | .78^‡^ |
| RV | 214 (21) | 37 (20) | 177 (21) | .87 |
| Other pathogens**^††^** | 237 (23) | 38 (21) | 199 (24) | .44 |
| Clinical course |  |  |  |  |
| Positive pressure ventilation use^‡‡^ | 55 (5) | 10 (5) | 45 (5) | 1.00 |
| Intensive treatment use^§§^ | 163 (16) | 26 (14) | 137 (16) | .55 |

Note: Data are the number (percentage) of children unless otherwise indicated. Percentages may not equal 100 because of rounding and missingness. All data are collected, unless otherwise indicated.

Abbreviations: IgE, immunoglobulin E; IQR, interquartile range; RSV, respiratory syncytial virus; RV, rhinovirus; tIgE, total immunoglobulin E.

**^†^** Tested by the chi-square test, unless otherwise indicated.

^‡^ Tested by the Wilcoxon rank sum test.

^§^ Defined as the use of inhaled and/or systemic corticosteroids before the index hospitalization.

# Table E4. Baseline patient characteristics and clinical course of infants hospitalized for bronchiolitis in the analytic cohort, using for 5 phenotypes in the sensitivity analysis

| **Variables** | **Phenotype A**  **(Corresponding to phenotype 1)**  **(n = 47; 26%)** | **Phenotype B**  **(Corresponding to phenotype 2)**  **(n = 39; 21%)** | **Phenotype C**  **(Corresponding**  **to phenotype 3)**  **(n = 49; 27%)** | **Phenotype D**  **(Corresponding**  **to phenotype 4)**  **(n = 27; 26%)** | **Phenotype E**  **(Corresponding to phenotype 2 and 4)**  **(n = 20; 26%)** | ***P* value^†^** |
| --- | --- | --- | --- | --- | --- | --- |
| Demographics |  |  |  |  |  |  |
| Age (month), median (IQR) | 2 (1–4) | 2 (1–4) | 5 (2–7) | 4 (2–8) | 5 (3–8) | <.001^‡^ |
| Male sex | 27 (57) | 23 (59) | 24 (49) | 16 (59) | 14 (70) | .59 |
| Race/ethnicity |  |  |  |  |  | .001 |
| Non-Hispanic white | 23 (49) | 23 (59) | 21 (43) | 4 (15) | 4 (20) |  |
| Non-Hispanic black | 8 (17) | 4 (10) | 14 (29) | 13 (48) | 6 (30) |  |
| Hispanic | 56 (31) | 16 (34) | 9 (23) | 11 (22) | 10 (37) |  |
| Other | 6 (3) | 0 (0) | 3 (8) | 3 (6) | 0 (0) |  |
| C-section delivery | 18 (38) | 9 (23) | 20 (41) | 15 (32) | 15 (32) | .37 |
| Prematurity (32–36.9 weeks) | 9 (19) | 4 (10) | 11 (22) | 7 (15) | 7 (15) | .46 |
| History of eczema | 4 (9) | 2 (5) | 8 (16) | 10 (21) | 10 (21) | .10 |
| Previous breathing problems (count) |  |  |  |  |  | .025 |
| 0 | 18 (38) | 9 (23) | 20 (41) | 9 (33) | 6 (30) |  |
| 1 | 9 (19) | 4 (10) | 11 (22) | 6 (22) | 1 (5) |  |
| ≥2 | 4 (9) | 2 (5) | 8 (16) | 6 (22) | 4 (20) |  |
| Lifetime corticosteroid use^§^ | 7 (15) | 7 (18) | 6 (12) | 6 (22) | 5 (25) | .67 |
| Pre-hospitalization corticosteroid use^¶^ | 3 (6) | 6 (15) | 5 (10) | 3 (11) | 2 (10) | .76 |
| Ever attended daycare | 11 (23) | 12 (31) | 17 (35) | 7 (26) | 7 (35) | .74 |
| Cigarette smoke exposure at home | 8 (17) | 3 (8) | 7 (14) | 7 (26) | 2 (10) | .31 |
| Parental history of eczema | 7 (15) | 7 (18) | 12 (24) | 6 (22) | 4 (20) | .81 |
| Parental history of asthma | 16 (34) | 14 (36) | 17 (35) | 10 (37) | 7 (35) | 1.00 |
| Clinical presentation at index hospitalization |  |  |  |  |  |  |
| Weight (kg), median (IQR) | 5.2 (4.3–6.2) | 5.5 (4.4–7.0) | 7.2 (5.3–8.5) | 6.5 (4.5–8.2) | 7.2 (6.1–9.0) | <.001^‡^ |
| Respiratory rate (per minute), median (IQR) | 48 (40–56) | 48 (40–60) | 46 (38–60) | 49 (40–64) | 46 (42–58) | .87 ^‡^ |
| Oxygen saturation |  |  |  |  |  | .52 |
| <90% | 5 (11) | 2 (5) | 2 (4) | 1 (4) | 1 (5) |  |
| 90–93.9% | 6 (13) | 5 (13) | 8 (16) | 0 (0) | 1 (5) |  |
| ≥94% | 36 (77) | 31 (79) | 38 (78) | 25 (93) | 18 (90) |  |
| Blood testing |  |  |  |  |  |  |
| Blood eosinophilia (≥4%) | 5 (11) | 3 (8) | 3 (6) | 3 (11) | 1 (5) | .85 |
| tIgE (kU/L), median (IQR) | 1.9 (1.9–2.7) | 1.9 (1.9–2.4) | 10.9 (6.6–22.4) | 17.4 (8.1–43.5) | 21.4 (10.6–39.2) | <.001^‡^ |
| Allergic (specific IgE) sensitization | 4 (9) | 2 (5) | 17 (35) | 13 (48) | 9 (45) | <.001 |
| Aeroallergen sensitization | 0 (0) | 0 (0) | 0 (0) | 1 (4) | 0 (0) | .22 |
| Food sensitization | 4 (9) | 2 (5) | 17 (35) | 12 (44) | 9 (45) | <.001 |
| Viral testing |  |  |  |  |  |  |
| RSV | 47 (100) | 35 (90) | 49 (100) | 27 (100) | 9 (45) | <.001 |
| RSV cycle threshold values | 21 (19–22) | 26 (23–27) | 21 (19–22) | 26 (25–30) | 23 (22–26) | <.001^‡^ |
| RV | 0 (0) | 17 (44) | 0 (0) | 0 (0) | 20 (100) | <.001 |
| Other pathogens**^††^** | 6 (13) | 7 (18) | 10 (20) | 6 (22) | 9 (45) | .057 |
| Clinical course |  |  |  |  |  |  |
| Positive pressure ventilation use^‡‡^ | 3 (6) | 4 (10) | 0 (0) | 2 (7) | 1 (5) | .31 |
| Intensive treatment use^§§^ | 26 (14) | 5 (11) | 6 (15) | 6 (12) | 7 (26) | .41 |

Note: Data are the number (percentage) of children unless otherwise indicated. Percentages may not equal 100 because of rounding and missingness. All data are collected, unless otherwise indicated.

Abbreviations: IgE, immunoglobulin E; IQR, interquartile range; RSV, respiratory syncytial virus; RV, rhinovirus; tIgE, total immunoglobulin E.

**^†^** Tested by the chi-square test, unless otherwise indicated.

^‡^ Tested by the Kruskal-Wallis test.

^§^ Defined as the use of inhaled and/or systemic corticosteroids before the index hospitalization.

^¶^ Defined as the use of inhaled and/or systemic corticosteroids for breathing problems that caused the index hospitalization.

**^††^** Adenovirus, bocavirus, *Bordetella pertussis*, enterovirus, human coronavirus NL63, OC43, 229E, or HKU1, human metapneumovirus, influenza A or B virus, *Mycoplasma pneumoniae*, and parainfluenza virus 1–3.

^‡‡^ Defined as the use of invasive and/or non-invasive mechanical ventilation (e.g., continuous positive airway pressure ventilation) during the index hospitalization.

^§§^ Defined as the use of positive pressure ventilation and/or admission to intensive care unit.

**Figure E1. Consensus matrices and cumulative distribution function plot to identify an optimal number of the virus clusters**

­­
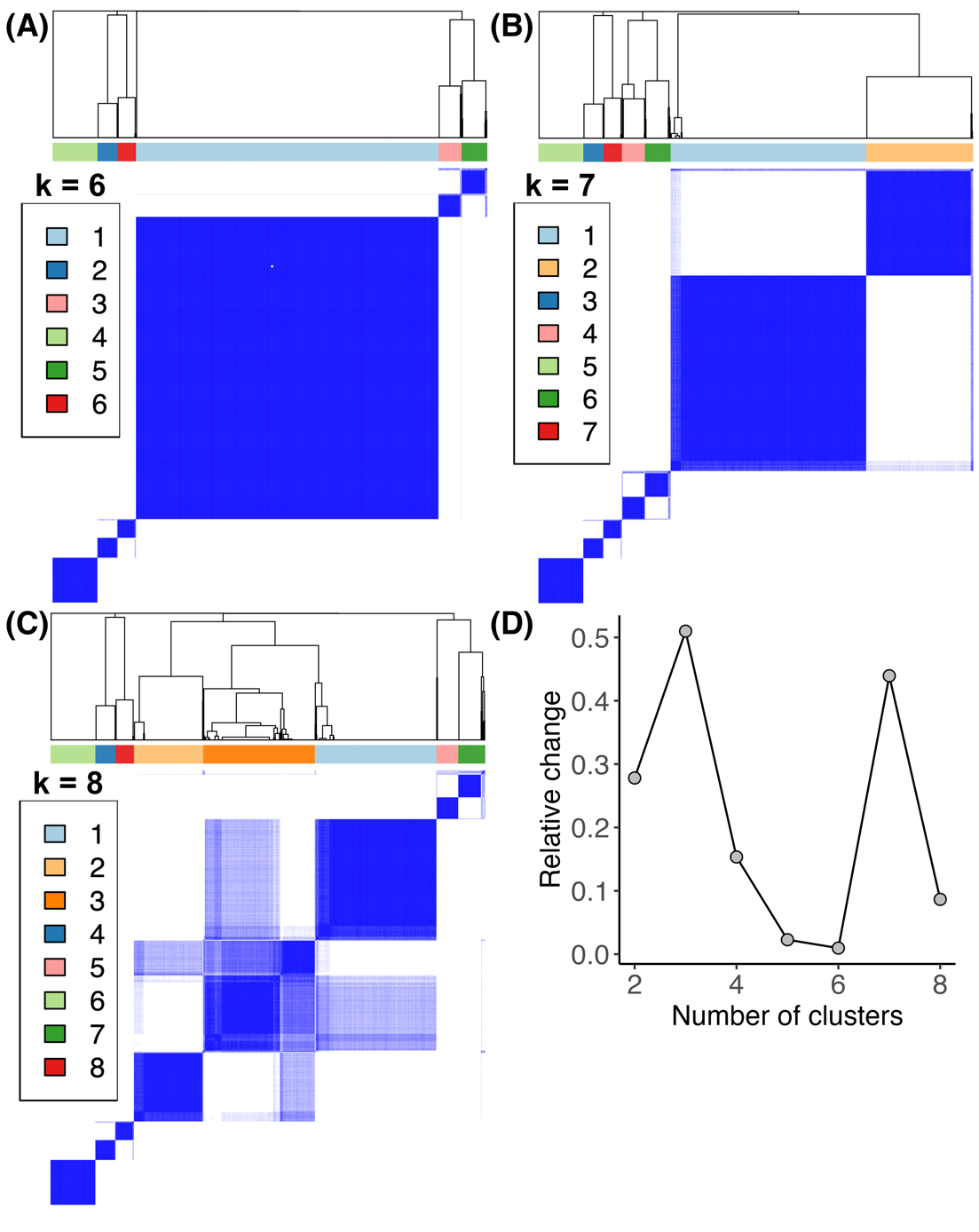


**(A-C)** The heatmaps visualize the consensus matrices for k = 6–8. The consensus matrix is obtained by taking the average over the connectivity matrices of every perturbed dataset. Consensus values range from 0 (never clustered together) to 1 (always clustered together) are marked by white to dark blue. It shows optimal partitioning in the consensus matrix with k = 7.

**(D)** The path plot visualizes the relative change in area under the cumulative distribution function (CDF) curve. Across the different numbers of clusters (k of 2–8), the relative change in the area under the CDF curve had little changes beyond k = 7.

**Figure E2. Consensus matrices and cumulative distribution function plot to identify an optimal number of the total immunoglobulin E-virus clusters**


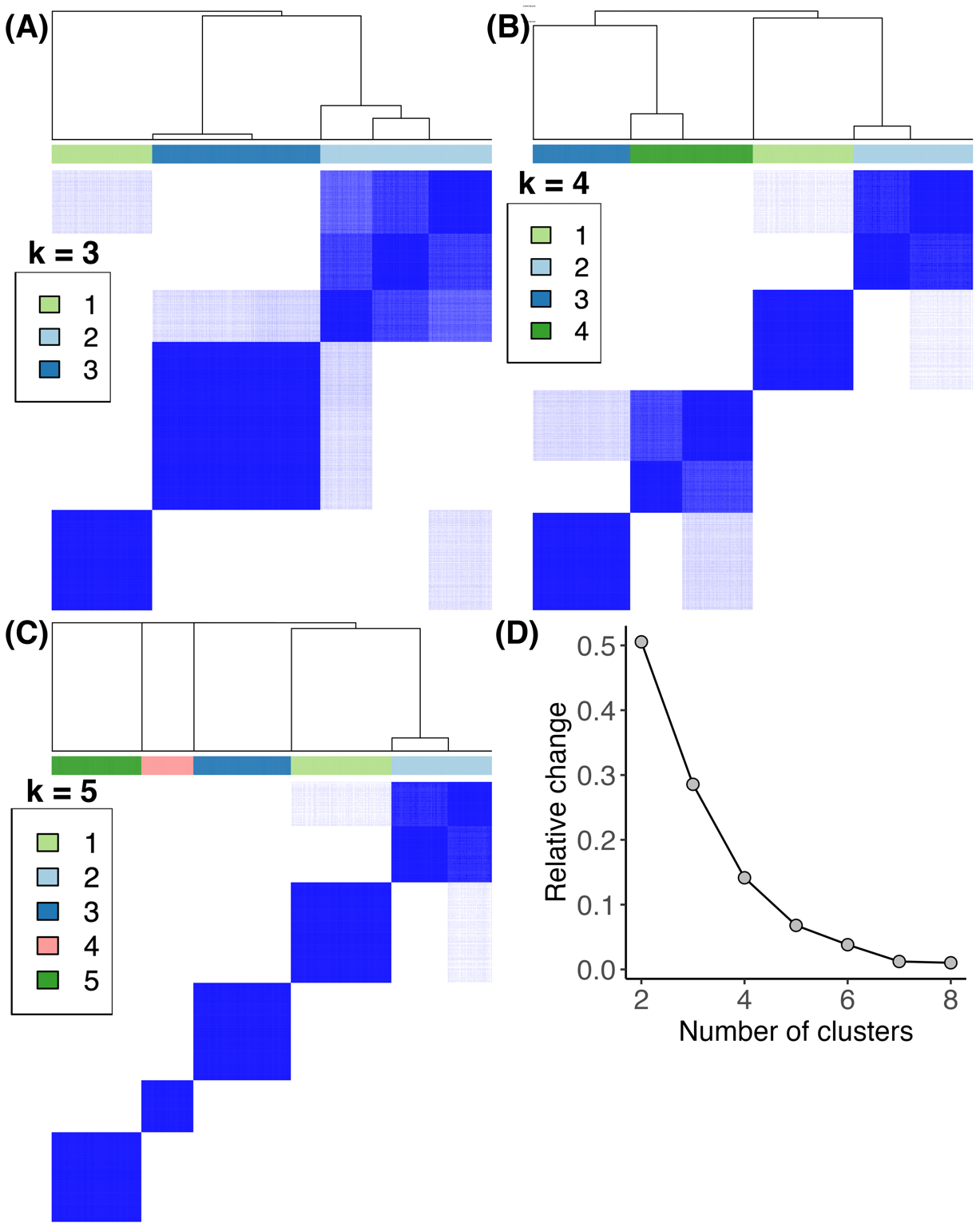


**(A-C)** The heatmaps visualize the consensus matrices for k = 3–5. The consensus matrix is obtained by taking the average over the connectivity matrices of every perturbed dataset. Consensus values range from 0 (never clustered together) to 1 (always clustered together) are marked by white to dark blue. It shows optimal partitioning in the consensus matrix with k = 4.

**(D)** The path plot visualizes the relative change in area under the cumulative distribution function (CDF) curve. Across the different numbers of clusters (k of 2–8), the relative change in the area under the CDF curve had little changes beyond k = 4.

**Figure E3. Differential gene expression analysis between phenotypes in infants hospitalized for bronchiolitis**


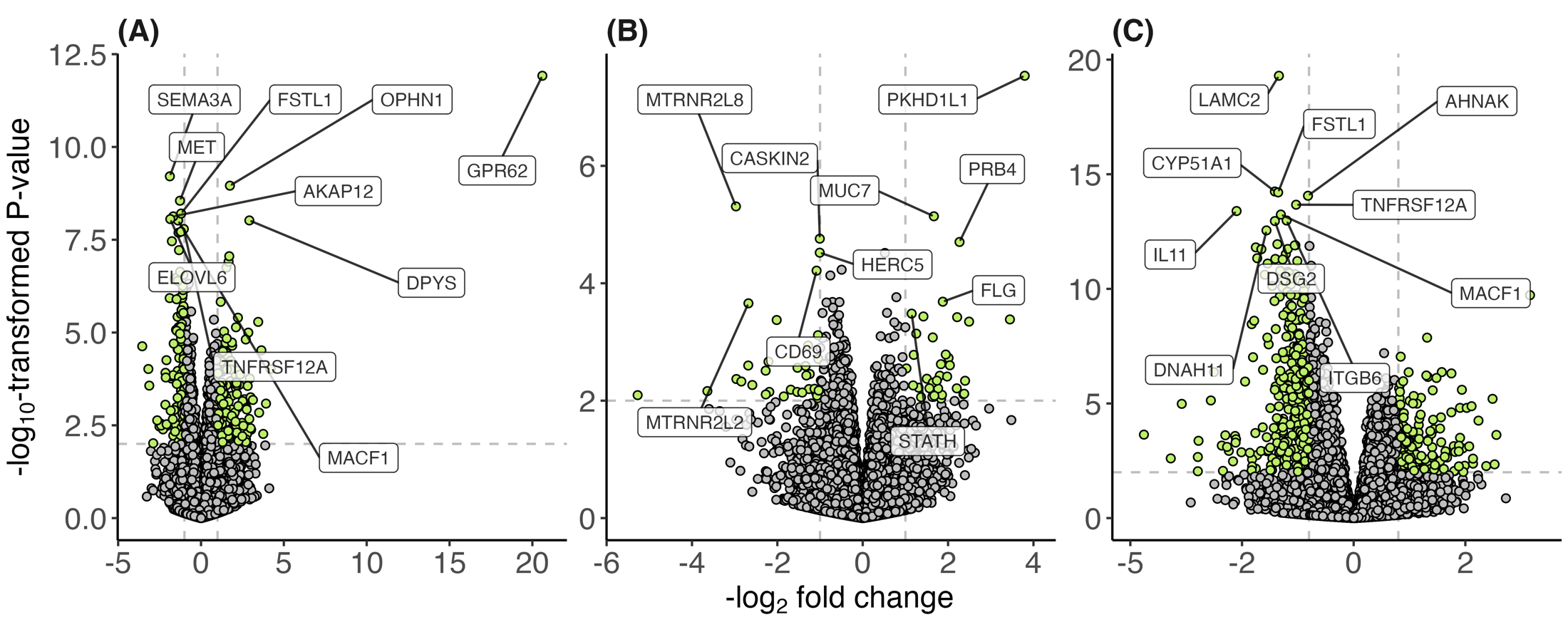


The volcano plots visualize differentially expressed genes between phenotype **(A)** 1 vs. 2 (phenotype 1 was set as the reference), **(B)** 1 vs. 3, and **(C)** 1 vs. 4. The threshold of log_2_ fold change is |1.0| shown by the vertical dashed line and that of *P* value is <.01 (i.e., >2 -log_10_-transformed *P* value) shown by the horizontal dashed line. Genes that satisfied these two thresholds were colored green, while others were colored gray. Of the green-colored gene, the 10 genes with the lowest *P* value (i.e., the highest -log_10_-transformed *P* value) were labeled.

**Figure E4. Between-phenotype difference (1 vs. 2) in nasopharyngeal mRNA pathways among infants hospitalized for bronchiolitis**

**
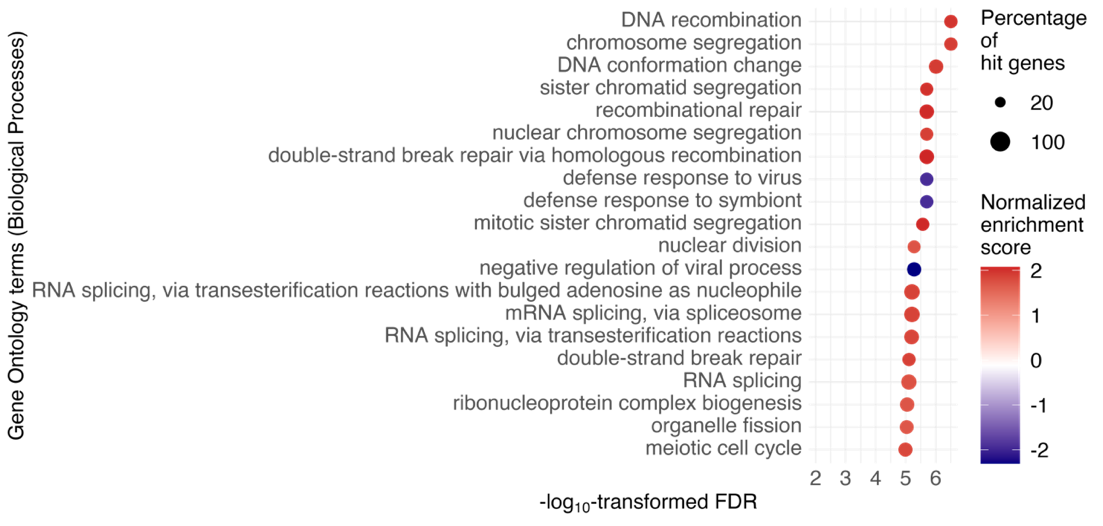
**

To examine the difference in the biological characteristics between phenotypes (1 [the reference] vs. 2), we applied the gene set enrichment analysis based on Biological Processes in Gene Ontology to the nasopharyngeal mRNA data. We identified enriched (normalized enrichment score [NES] ≥0 and false discovery rate [FDR] <.10) or depleted (NES <0 and FDR <.10) pathways. Of the identified pathways, we selected the 20 pathways with the lowest FDR.

**Figure E5. Differential microRNA expression analysis between phenotypes in infants hospitalized for bronchiolitis**


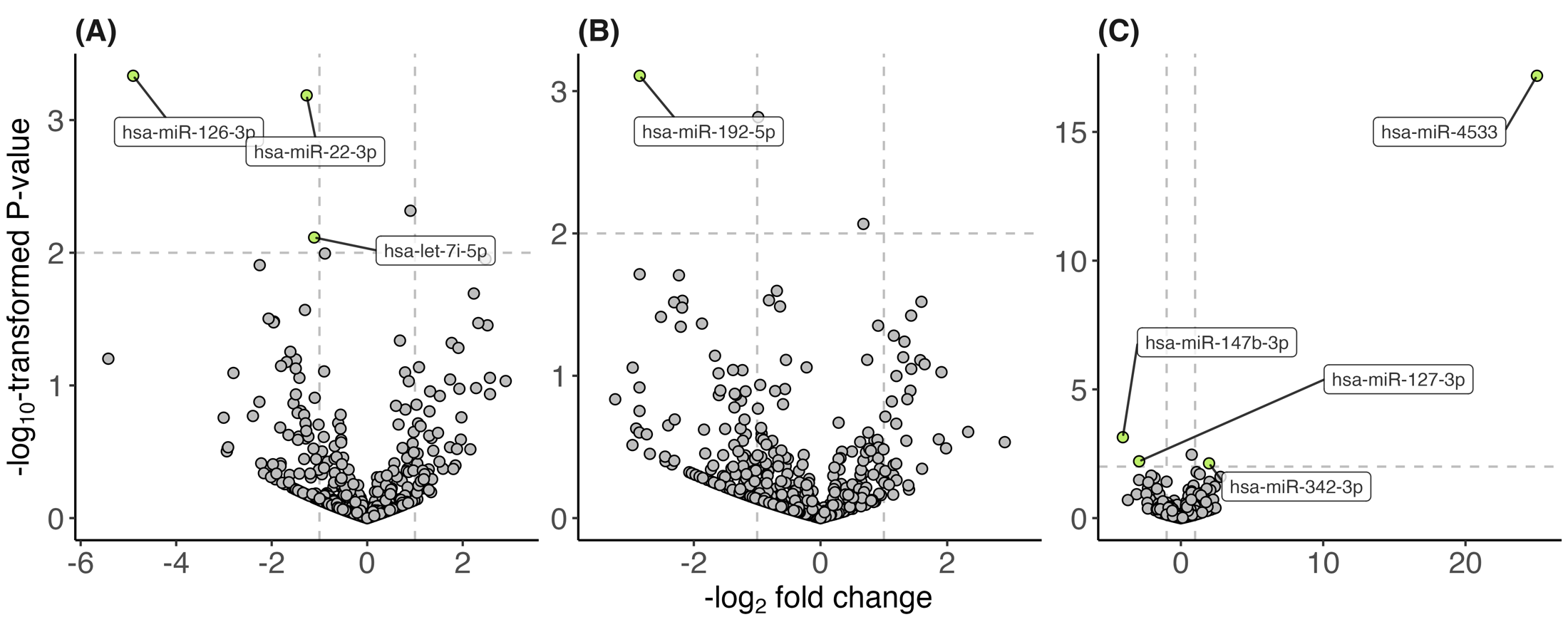


The volcano plots visualize differentially expressed microRNA (miRNA) between phenotype (**A)** 1 vs. 2 **(**phenotype 1 was set as the reference), **(B)** 1 vs. 3, and **(C)** 1 vs. 4. The threshold of log_2_ fold change is |1.0| shown by the vertical dashed line and that of *P* value is <.01 (i.e., >2 -log_10_-transformed *P* value) shown by the horizontal dashed line. miRNA that satisfied these two thresholds were colored green and labeled, while others were colored gray.

**Figure E6. Between-phenotype difference (1 vs. 2) in nasopharyngeal mRNA pathways and nasal microRNA pathways among infants hospitalized for bronchiolitis**

**
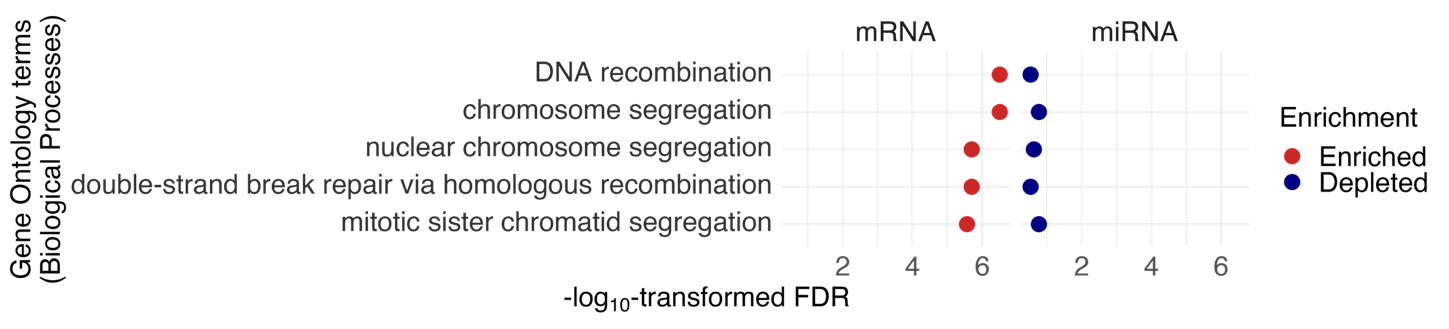
**

To examine the difference in the biological characteristics between phenotypes (1 [the reference] vs. 2), we performed the gene set enrichment analysis based on Biological Processes in Gene Ontology to the nasopharyngeal mRNA data and the nasal microRNA (miRNA) data. We identified the pathways “enriched in the mRNA data and depleted in the miRNA data” or “depleted in the mRNA and enriched in the miRNA data” (false discovery rate [FDR] <.10), accounting for suppressive nature of miRNA to mRNA.

**Figure E7. Association of phenotypes of infant bronchiolitis with risk of developing childhood asthma, limiting to infants without a previous history of breathing problems in the sensitivity analysis.**


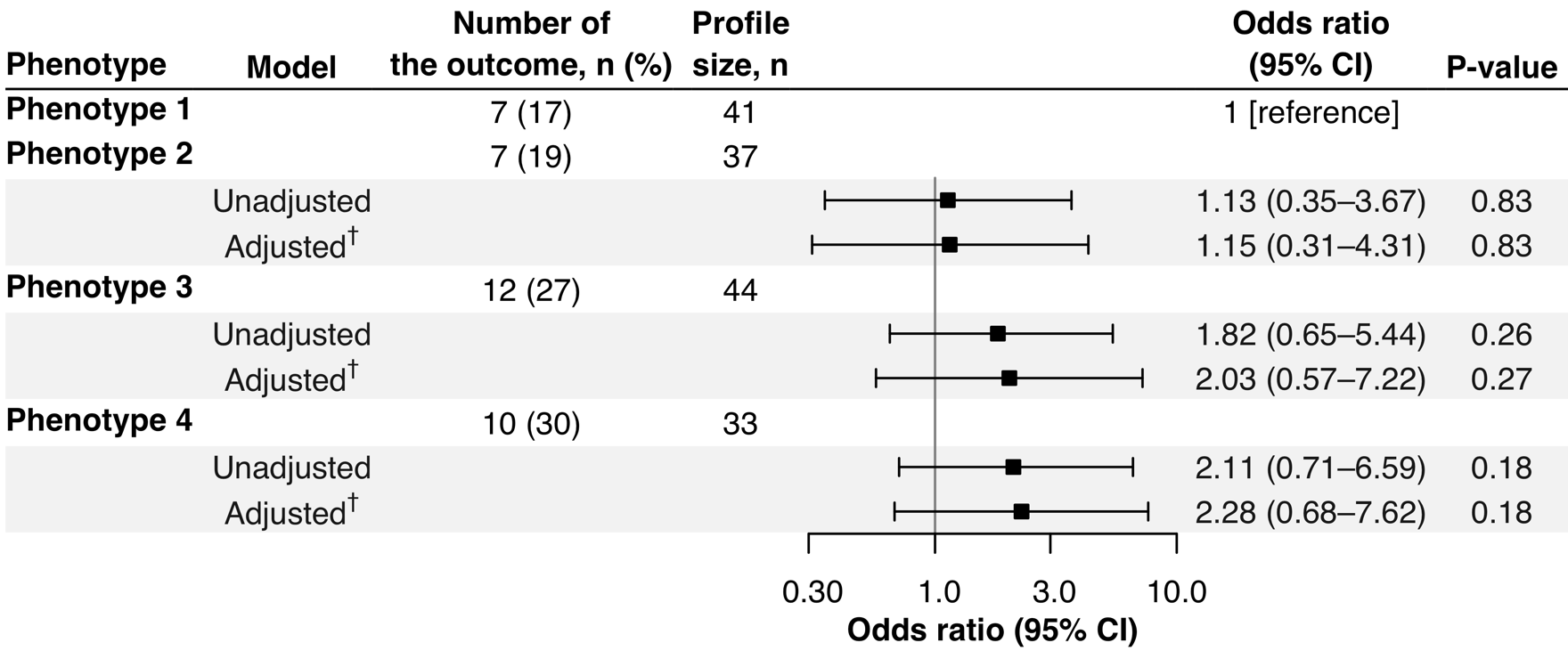


To examine the association of bronchiolitis phenotypes (phenotype 1 as the reference) with the risk of developing asthma, logistic regression models were constructed.

^†^ Multivariable mixed-effects logistic regression model accounting for patient clustering by site and adjusted for potential confounders (i.e., age, sex, parental history of asthma, prematurity [<37 weeks], and pre-hospitalization use of inhaled and/or systemic corticosteroids).

# Figure E8. Alluvial plot to examine consistencies across different numbers of phenotypes


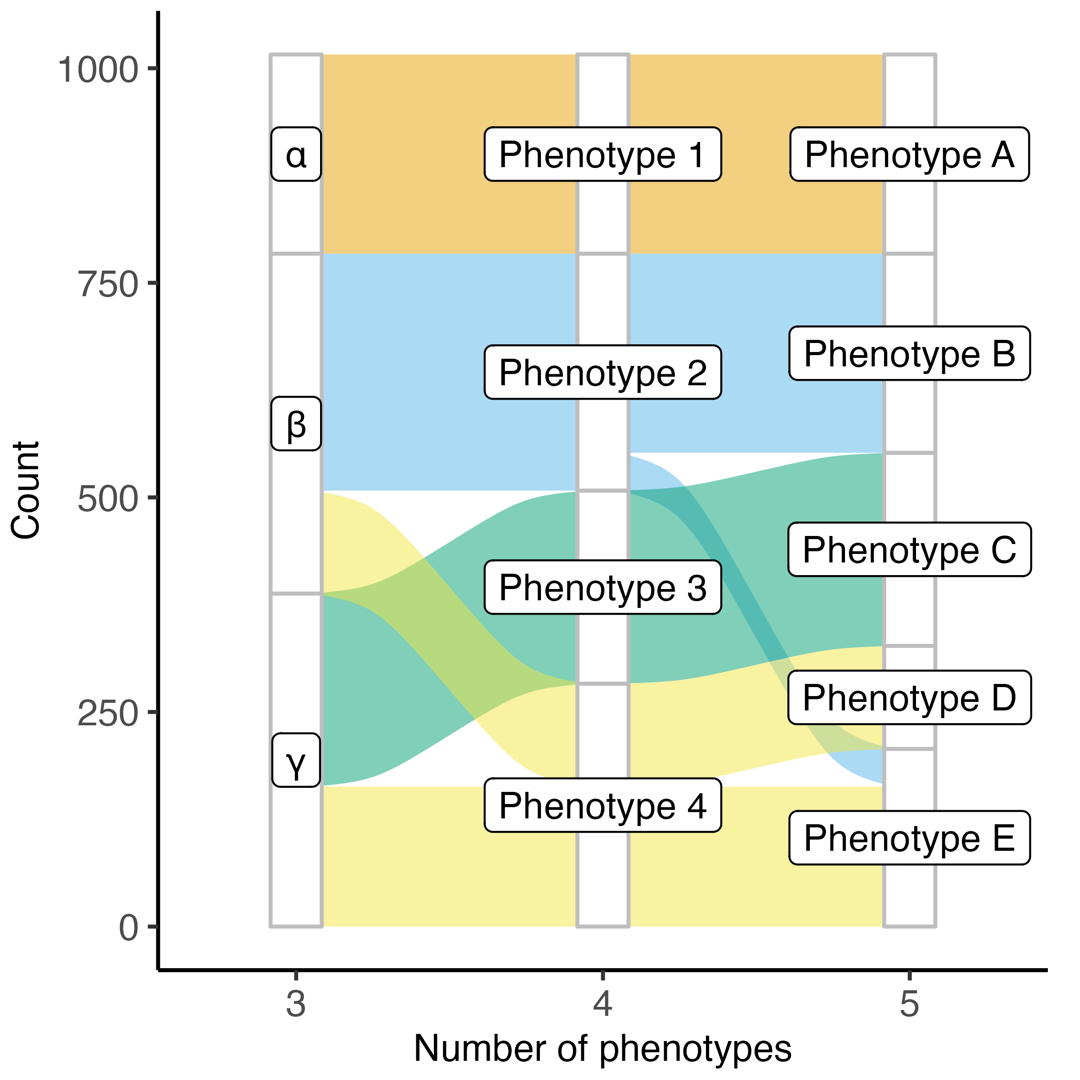


The *X* axis indicates the number of phenotypes (range of 3–5); the *Y* axis indicates the count of infants. Each color band represents a group of infants with an original phenotype (1–4). Consistencies were confirmed between the 4 original phenotypes (1–4) and 3 and 5 phenotypes.

**Figure E9. Clinical and virus characteristics of infants hospitalized for bronchiolitis, according to phenotypes, by using for 5 phenotypes in the sensitivity analysis**


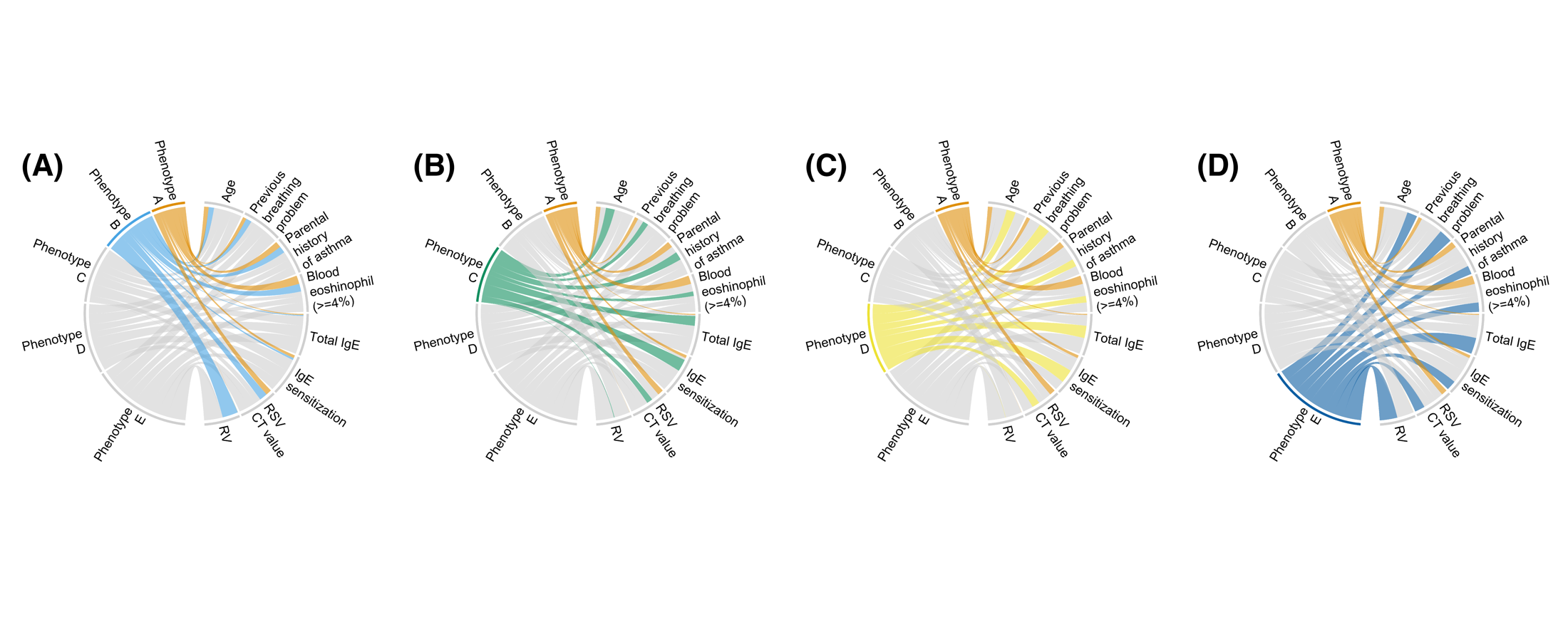


To interpret clinical and virus characteristics of the 5 phenotypes, we constructed chord diagrams that represent the comparison between phenotypes **(A)** A and B, **(B)** A and C, **(C)** A and D, and **(D)** A and E. Ribbons connect each of the phenotypes (phenotypes A–E) with major clinical and virus characteristics. The width of the ribbon represents the proportion of infants or the mean value within the phenotypes who have the corresponding clinical or virus characteristic, which was scaled to a total of 100% or 1. Of the clinical and virus characteristics, we selected 8 characteristics that may be related to “classic” bronchiolitis and coordinated the presence or increase of those in the same direction. Hence, the phenotype with the narrower bundle of ribbons resembles “classic” bronchiolitis (phenotype A).

Abbreviations: CT, cycle threshold; IgE, immunoglobulin E; RSV, respiratory syncytial virus; RV, rhinovirus.

**Figure E10. Association of phenotypes of infant bronchiolitis with risk of developing childhood asthma, using 5 phenotypes in the sensitivity analysis**


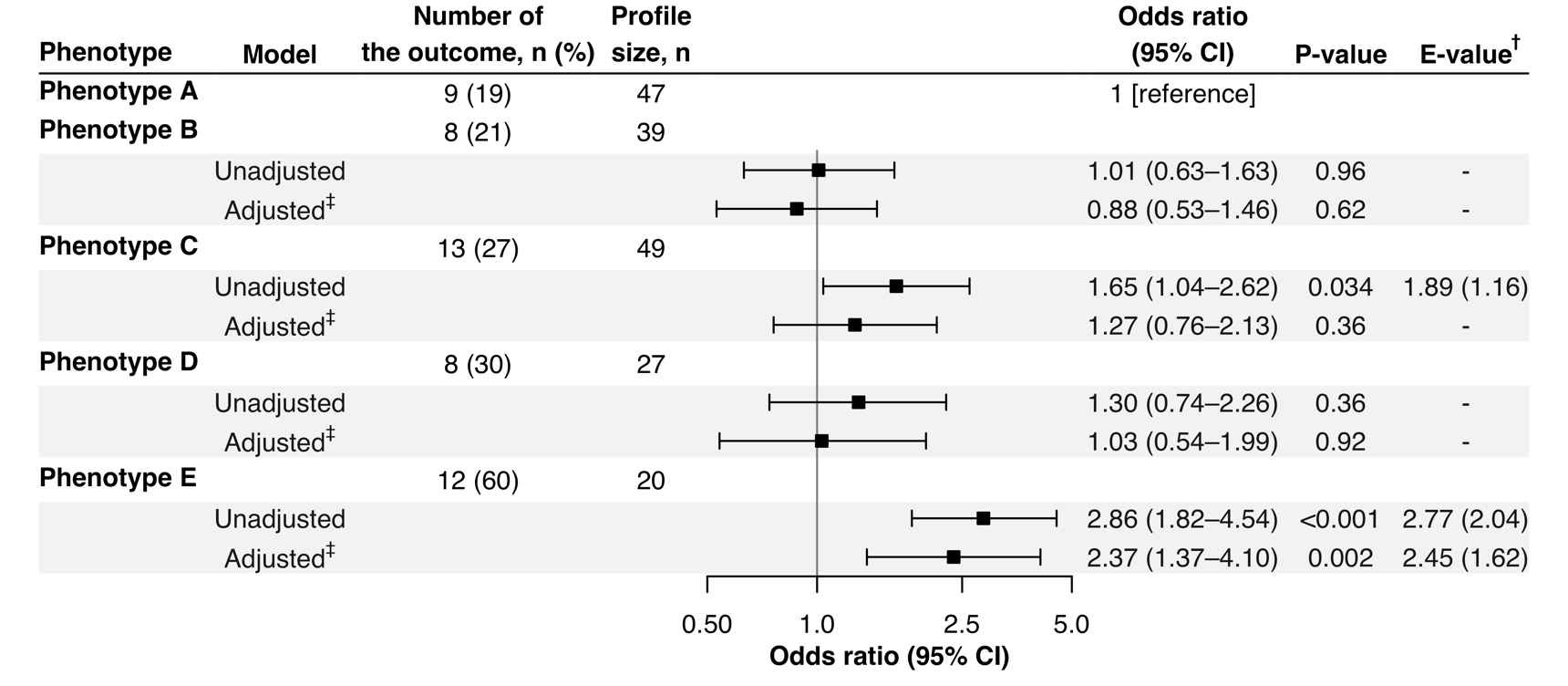


To examine the association of bronchiolitis phenotypes (phenotype A as the reference) with the risk of developing asthma, logistic regression models were constructed.

^†^ The E-value (with its lower 95% confidence interval [CI] bound) represents how strongly unmeasured confounder(s) represents how strongly a set of unmeasured confounders would be associated with the exposure and outcome to fully eliminate the observed association.

^‡^ Multivariable mixed-effects logistic regression model accounting for patient clustering by site and adjusted for potential confounders (i.e., age, sex, parental history of asthma, prematurity [<37 weeks], previous history of breathing problems, and pre-hospitalization use of inhaled and/or systemic corticosteroids).
